# Research hotspots and trend analysis of abdominal pain in inflammatory bowel disease: a bibliometric and visualized analysis

**DOI:** 10.3389/fphar.2023.1220418

**Published:** 2023-09-21

**Authors:** Shuai Peng, Yuan Xia, Ying Wang, Xiaoyun Yu, Zunan Wu, Li Zhang, Ke Xu, Lei Shen, Hesheng Luo

**Affiliations:** ^1^ Department of Gastroenterology, Renmin Hospital of Wuhan University, Wuhan, China; ^2^ Hubei Key Laboratory of Digestive Diseases, Renmin Hospital of Wuhan University, Wuhan, China; ^3^ Department of Gastroenterology, Union Hospital, Tongji Medical College, Huazhong University of Science and Technology, Wuhan, China; ^4^ Department of Neurology, Renmin Hospital of Wuhan University, Wuhan, China

**Keywords:** inflammatory bowel disease, abdominal pain, bibliometric, visceral hypersensitivity, citespace

## Abstract

**Aims:** The study aimed to provide a bibliometric and visual analysis of research on abdominal pain in inflammatory bowel disease and discuss the current status, research hotspots, and future developments.

**Methods:** We used the Web of Science Core Collection to comprehensively search the literature on abdominal pain-related research in IBD published between 2003 and 2022. The bibliometric and visual analysis was performed through CiteSpace, VOSviewer software, R language, and the bibliometric online analysis platform, including authors, institutions, countries, journals, references, and keywords in the literature.

**Results:** A total of 3,503 relevant articles are included, indicating that the number of articles in this field has increased in recent years. The United States leads the way with a dominant position in terms of article output, followed by China and JAPAN. United States (967 articles), University of Calgary (98 articles), and World Journal of Gastroenterology (127 articles) are the top publishing countries, institutions, and journals, respectively; keyword analysis shows that gut microbiota, depression, stress, visceral hypersensitivity, and multidisciplinary approach are the hot spots and trends in this research area.

**Conclusion:** Abdominal pain-related studies in IBD have received increasing attention in the past two decades. This study provides the first bibliometric analysis of papers in this research area using visualization software and data information mining. It provides insights into this field’s current status, hot spots, and trends. However, many outstanding issues in this research area still need further exploration to provide a theoretical basis for its clinical application.

## 1 Introduction

Inflammatory bowel disease (IBD) is a persistent and recurring inflammatory disorder affecting the gastrointestinal tract, encompassing ulcerative colitis (UC) and Crohn’s disease (CD). The etiology of this disease remains uncertain and is believed to result from a multifactorial interplay of genetic, immunological, and environmental factors primarily affecting young and middle-aged individuals (Bisgaard et al., 2022). Manifestations of IBD are diverse, often accompanied by symptoms of gastrointestinal dysfunction, including abdominal discomfort, diarrhea, and various extra-intestinal indications. Diagnosis entails a clinical evaluation integrating endoscopy and blood tests, with treatment strategies tailored to the severity of symptoms and patient-specific considerations (Rogler et al., 2021).

The presence of abdominal pain is a commonly reported symptom in individuals diagnosed with inflammatory bowel disease. Its pathogenesis is attributed to various factors, including intestinal inflammation, visceral hypersensitivity, psychological and sociological factors, and genetic factors predisposition (Bakshi et al., 2021). Epidemiological data have demonstrated that most patients diagnosed with active inflammatory bowel disease experience abdominal pain, frequently subsides with reduced disease activity. However, despite alleviating intestinal inflammation, some patients, exceeding 20%, experience symptoms akin to irritable bowel syndrome, such as visceral hypersensitivity and abdominal discomfort. This persistent pain is not merely a sensory output of bodily organs. However, it is somewhat correlated with pain processing and sensory pathways moderated by psychological symptoms such as stress, anxiety, depression, medication usage, sleep disturbances, and cognitive factors. The impact of persistent abdominal pain in this population is significant, impairing patients’ quality of life and contributing to the economic burden of the disease (Norton et al., 2017; Wils et al., 2022).

Over the past few years, a surge in research related to abdominal pain in IBD has resulted in a commensurate increase in literature on the subject. However, a systematic investigation into the field using bibliometric techniques to gain a deeper comprehension of the literature and discern research hotspots and trends has yet to be undertaken. Bibliometrics, a mathematical and statistical body of knowledge that intertwines with a bibliography, offers a comprehensive analytical approach to research literature quantification (Deng et al., 2022; Guan et al., 2022). The approach provides detailed information on authors, keywords, countries, institutions, journals, and references which are integral in reflecting the features and future directions of the research field. The amalgamation of bibliometric and visual analysis techniques such as CiteSpace, VOSviewer, and R language has recently facilitated a more vivid and intuitive representation of literary data, enabling the expeditious identification of patterns and trends, enhancing analytical efficiency, and heightening research authenticity (Peng et al., 2022; Wan et al., 2022).

Therefore, this paper aims to explore the research hotspots and trends in abdominal pain in IBD research between 2003 and 2022 based on bibliometric and visual analyses, evaluate the quality and quantity of academic results in this field, and improve the efficiency and scientific research of researchers. Furthermore, the study will deepen our understanding of abdominal pain in IBD research and promote the formulation of more targeted research strategies.

## 2 Materials and methods

### 2.1 Data source and search strategy

**FIGURE 1:**
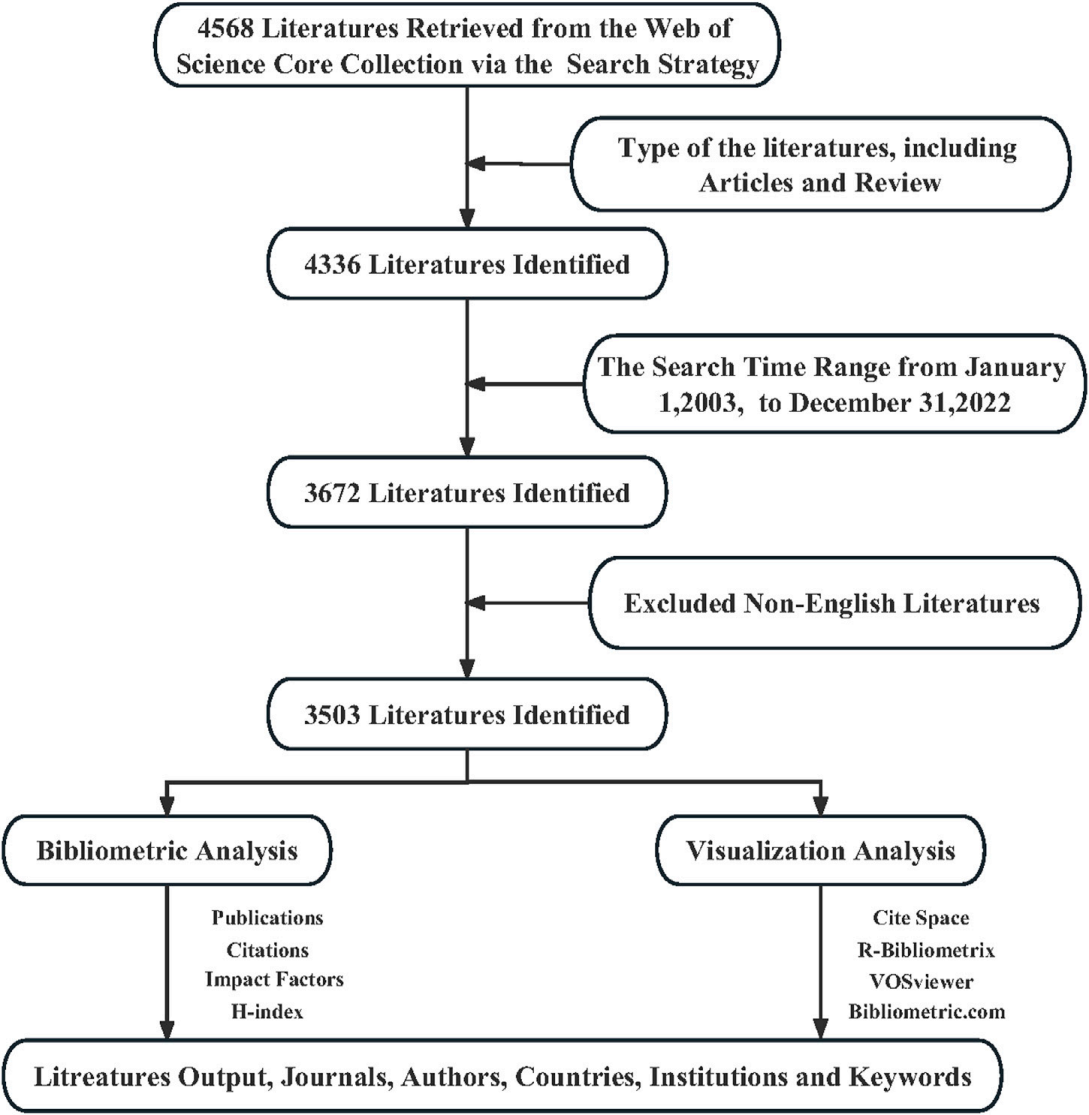
The flow chart of literature research, bibliometric analysis, and visualization analysis.

The Web of Science (WOS) constitutes a comprehensive and authoritative bibliographic database with broad coverage, exceptional quality, and rich metrics. It has gained considerable usage in bibliometric research (Li et al., 2018). Thus, the present study selected the Web of Science Core Collection (WoSCC) as the primary data source, utilizing a search period spanning from January 2003 to December 2022 and employing a search strategy consisting of the expression TS= (Inflammatory bowel diseases OR Ulcerative colitis OR colitis OR Crohn Disease) AND (Abdominal Pain OR “Pain, Abdominal” OR visceral pain). Figure 1 describes the specific search and analysis process. The search was conducted on 25 April 2023, identifying 4,568 articles after two independent researchers carefully screened titles, abstracts, and keywords. The study further restricted the literature type to Articles and Reviews and the language type to English, ultimately resulting in the selection of 3,503 articles for analysis. Any conflicting opinions arising between the two researchers during the screening process were resolved through consensus or referred to a third researcher.

### 2.2 Data processing and analysis

The data obtained from Web of Science was exported in plain text under the “Full Record and Cited References” option and subsequently imported into R version 4.2.3, CiteSpace version 6.2.R2, VOSviewer and the bibliometric online analysis platform (http://bibliometric.com/) for bibliometric and visual analysis. The data was meticulously analyzed using the bibliometrix package available in R to examine pertinent metrics such as authorship, institutional affiliation, country of origin, subject matter, number of publications, citation relationships, and other relevant factors. The analysis results were then presented using various visually engaging charts, such as word clouds, temporal graphs, and relationship diagrams. To assess the quality and standard of the journals, we also obtained the impact factor (IF) and JCR division of journals from the Journal Citation Reports (JCR).

The knowledge mapping tools, CiteSpace and VOSviewer, respectively, offer complementary strengths in bibliometric analysis. CiteSpace, developed by Professor Chaomei Chen of Drexel University, United States, features diverse analytical capabilities, user-friendly operation, and continual updates, making it a valuable tool for gaining deeper insights into the workings of research literature (Wang et al., 2022). For our research, we appropriately specified the parameters in CiteSpace to produce co-occurrence visualization maps of journals, authors, institutions, countries, references, and keywords. Furthermore, we conducted cluster analysis and emergence analysis of pertinent keywords. The software settings were as follows: the timespan was set as 2003–2022 (slice length = 1), while other parameters remained at their default values. In contrast, VOSviewer applies a probabilistic-based normalization approach and provides multiple visualization approaches for exploring keywords, co-authorship relationships, co-institutions, and other relevant research entities, with the added benefits of user-friendly mapping and visually appealing images (Peng et al., 2022). In addition, the bibliometric online analysis platform (http://bibliometric.com/) enables comprehensive analyses of total literature and co-authorship relationships and examines scientific disciplines and journals.

## 3 Results

### 3.1 Literature output and general characteristics

**FIGURE 2:**
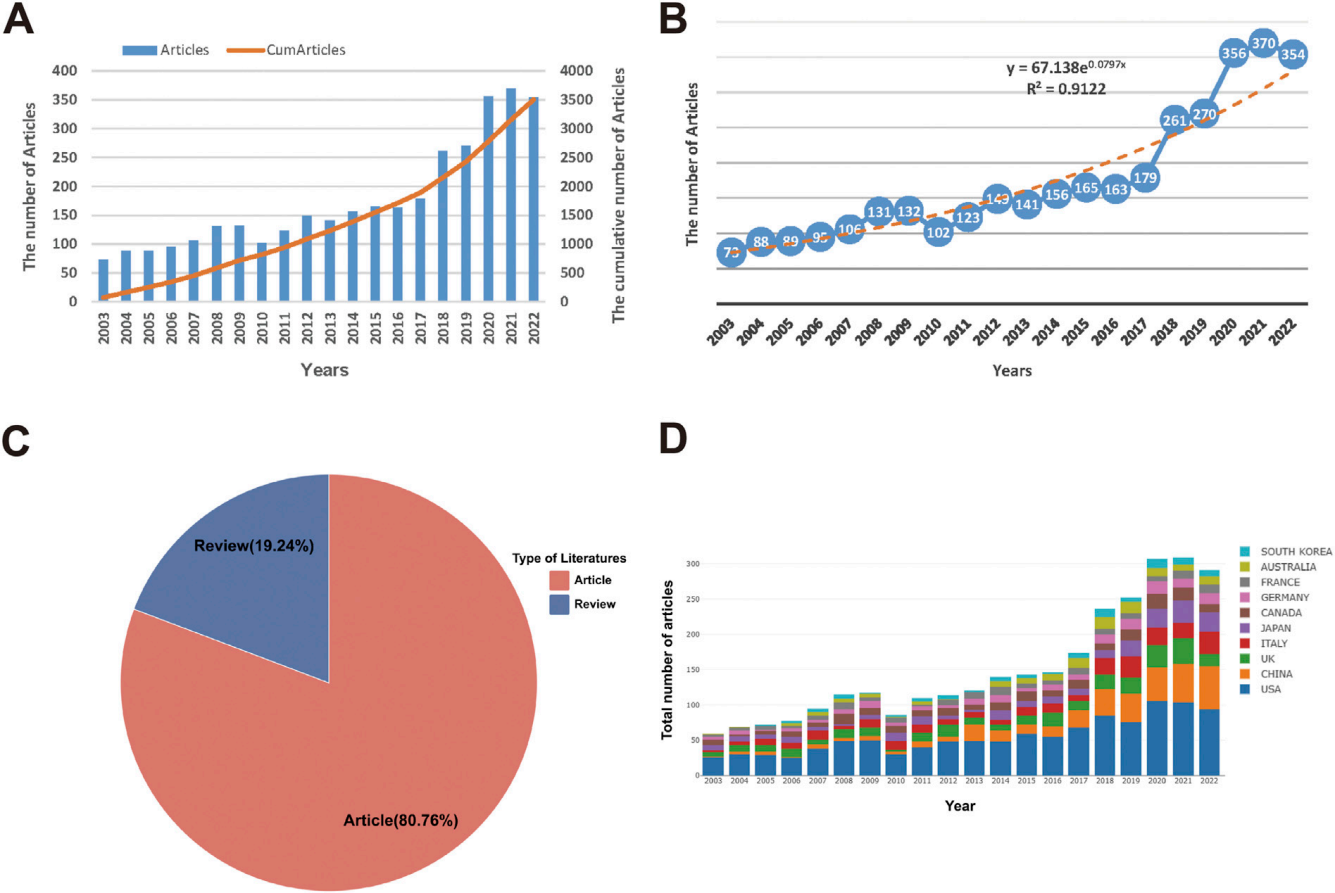
Literature output and general characteristics in the field of abdominal pain in IBD in the past two decades. The number and cumulative number of publications **(A)**, exponential growth graph of the number of publications **(B)**, literature type distribution **(C)**, and the total number of publications per year in common countries **(D)** in the field of abdominal pain in IBD in the past two decades.

The annual variation in the quantity of published literature may indicate research activity and development trends in a particular field. Based on our search results, the number of research publications concerning abdominal pain within the IBD research domain has been observed to increase consistently between 2003 and 2022. The cumulative annual publication count has demonstrated a steady and rapid upward trend, implying that abdominal pain is receiving growing attention in IBD research and are gradually emerging as a new research hotspot (Figure 2A). Applying Price’s law illustrates that the logistic regression model’s simulation of the literature growth trend fits well with an exponential growth curve, providing compelling evidence of a significant annual increase in publication numbers concerning abdominal pain in IBD research (*R* ^2^ = 0.9122) (Figure 2B). The majority of published literature identified in this study falls into the category of articles (Figure 2C). Additionally, utilizing bibliometric online analysis platforms, we have calculated the yearly frequency of publications from common publishing countries (Figure 2D).

### 3.2 Analysis of authors and co-authorship

**FIGURE 3:**
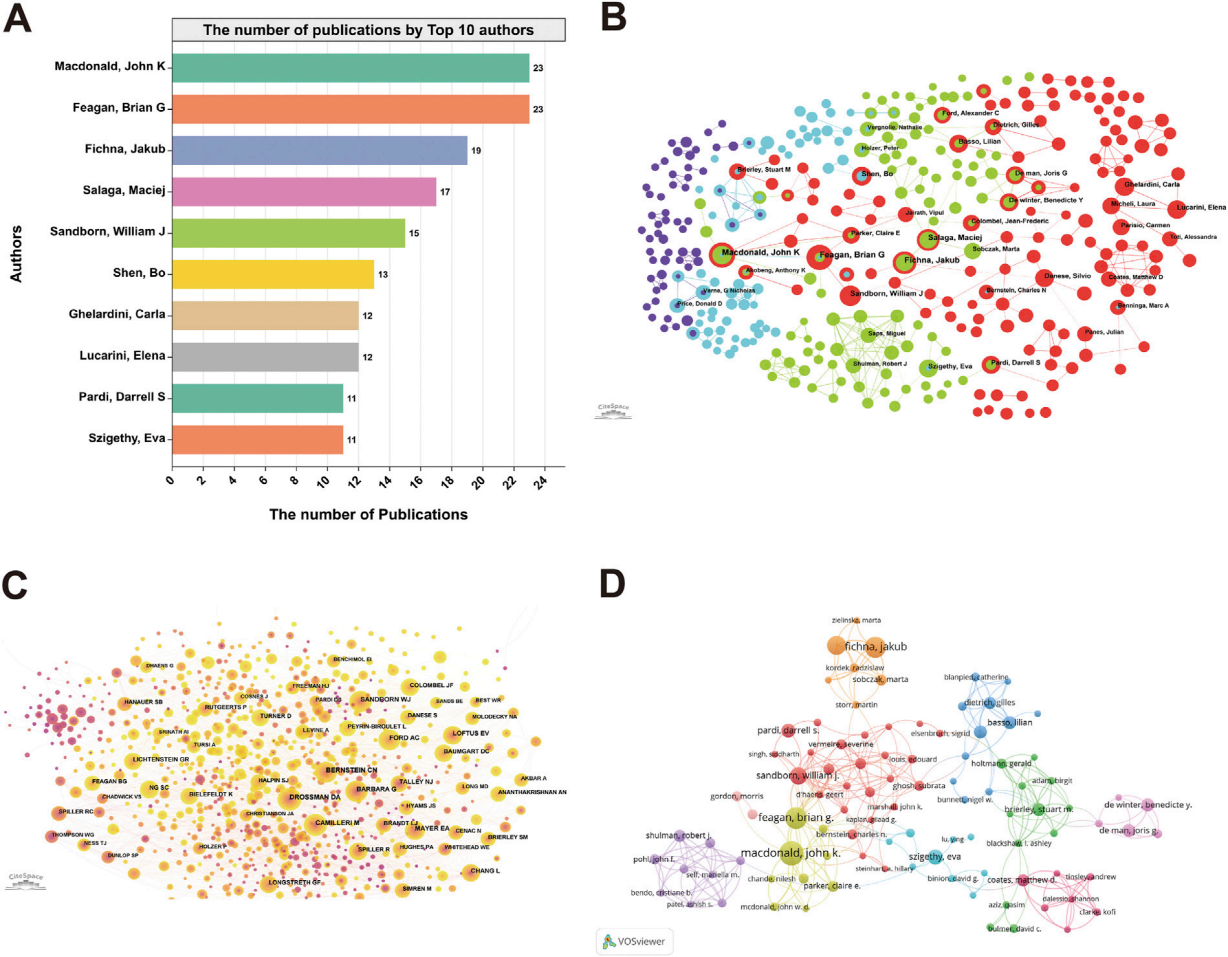
Visualization of authors and co-authorship related to the research on abdominal pain in IBD in the past two decades. The number of publications by Top 10 authors **(A)**, the co-occurrence of authors **(B)**, the co-occurrence of cited authors **(C)** and the cooperation relationship between authors **(D)** in the field of abdominal pain in IBD in the past two decades.

A comprehensive analysis of the literature authors has been undertaken to gain valuable insights into the scholars and key research competencies associated with the relevant field. The analysis reveals that out of 18,791 authors associated with the 3,503 articles published in the last two decades, the top 10 contributed 156 articles, accounting for 4.5% of the complete published works. Notably, MacDonald, John K contributed the highest number of articles (23, 0.65%), followed by Feagan, Brian G., Fichna, Jakub, Salaga, Maciej, and Sandborn, William J. (Figure 3A). Based on the Web of Science, the Highly Cited Index (H-index) is a hybrid quantitative index that can assess the volume and level of scholarship researchers produce. Among the authors, Feagan, Brian G. has the highest H-indexes. In addition, Sandborn, William J. ranks highest in average citation rate with 166.33 citations per paper, followed by Feagan, Brian G. (113.83) and Szigethy, Eva (45.27) (Table 1).

**TABLE 1:** Top 10 authors with the most publications.

Top 10 authors with the most publications						
Authors	Affiliation*	Publications	H-index	G-index	Citations	Average citation/Publication
MacDonald, John K	Alimentiv Inc	23	18	23	908	39.48
Feagan, Brian G	Alimentiv Inc	23	19	25	2,618	113.83
Fichna, Jakub	Medial Univ Lodz	19	15	22	571	30.05
Salaga, Maciej	Medical University Lodz	17	14	19	487	28.65
Sandborn, William J	University of California San Diego	15	17	21	2,495	166.33
Shen, Bo	Columbia University	13	10	16	334	25.69
GHELARDINI C	University of Florence	12	6	9	86	7.17
Lucarini, Elena	University of Florence	12	6	9	86	7.17
Pardi, Darrell S	Mayo Clinic	11	10	14	390	35.45
Szigethy, Eva	University of Pittsburgh	11	12	13	498	45.27

The present study utilizes CiteSpace to construct a visual map of co-occurrence to understand the author comprehensively and cited collaboration relationships. Over the past 20 years, the author collaboration network comprised 329 nodes, while the cited author collaboration network encompassed 951. Each node represents a particular author, with node size denoting the number of co-authored papers and connecting lines signifying collaborative relationships between authors. The same color is assigned to authors in the same cluster. The larger the author group node, the greater the number of co-authored articles (Figures 3B,C). Moreover, VOSviewer was employed to visualize and analyze the collaboration graph between authors who published more than five papers. The collaboration network can be divided into ten primary clusters of authors. The red cluster has the highest number of collaborators, such as Sandborn, William J., Pardi, Darrell S., Vermeire, Severine, and Bernstein, Charles N. The green and blue clusters also demonstrate collaborative relationships within the clusters, forming stable research teams (Figure 3D).

### 3.3 Analysis of the countries

**FIGURE 4:**
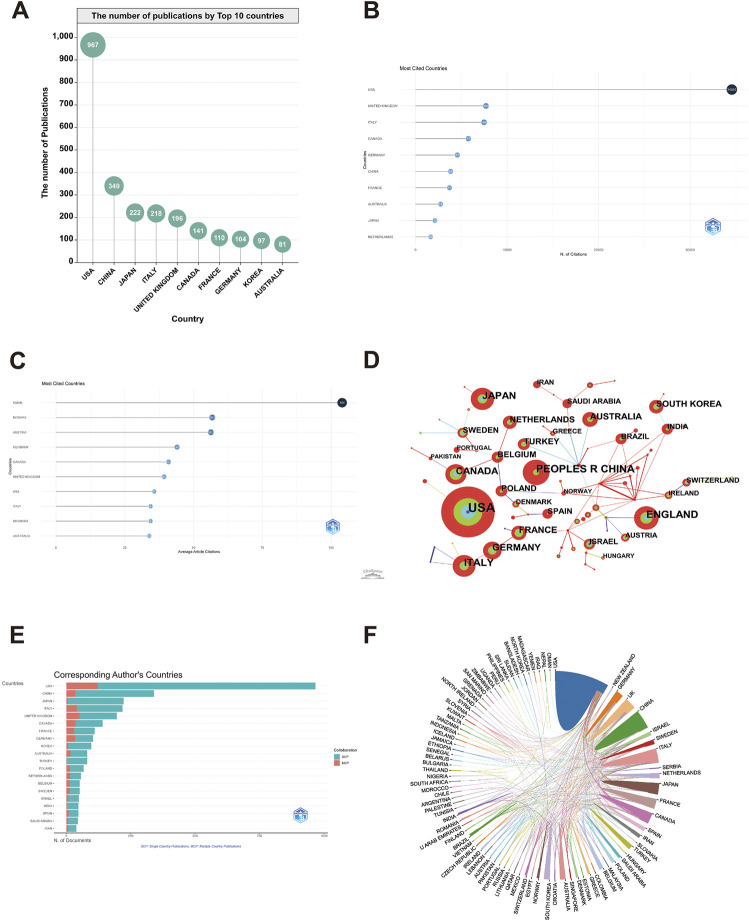
Contributions of various countries to the research of abdominal pain in IBD in the past two decades. The number of publications by Top 10 countries **(A)**, the Top 10 cited countries ranked by total citations **(B)**, the Top 10 cited countries ranked by average citations **(C)**, the co-occurrence of countries **(D)**, the corresponding author’s countries **(E)**, and the cooperation relationship between countries **(F)** in the field of abdominal pain in IBD in the past two decades.

Our search findings indicate that abdominal pain has been studied in the context of IBD research by scholars hailing from 597 countries and regions during the previous two decades. Our analysis further reveals that the United States, with 967 publications, held the top position, followed by China (340), Japan (222), Italy (218), and United Kingdom (196) (Figure 4A). Bibliometric assessed countries’ contribution to abdominal pain and IBD research and illustrated it in the form of a global map, with China and the United States found to be the leading players involved in this research area significant players in this research area (Supplementary Figure S1, S2). The United States had the most cited studies (34,543), followed by the United Kingdom (7,695), Italy (7,489), Canada (5,770), and Germany (4,574) (Figure 4B). Subsequent analysis, focused on rank by average citation rate, revealed that Oman (average citation rate = 104), Norway (average citation rate = 56.8), and Australia (average citation rate = 56.3) were the top three countries in this regard (Figure 4C).

The present study employed CiteSpace to analyze the countries’ collaborative networks and generate a co-occurrence map. Over the previous two decades, 101 countries or regions collaborated on publications, with United States emerging as the most collaborative country, followed by China, Japan, and Italy (Figure 4D). However, an analysis of the Multiple Country Publications (MCP) rates reveals that international cooperation is more frequent in the United States and the United Kingdom (Figure 4E). Furthermore, the study utilized Bibliometrics Online analysis to investigate country-to-country collaboration further, revealing a dearth of collaborative efforts with a partial exception (Figure 4F).

### 3.4 Analysis of the institutions

**FIGURE 5:**
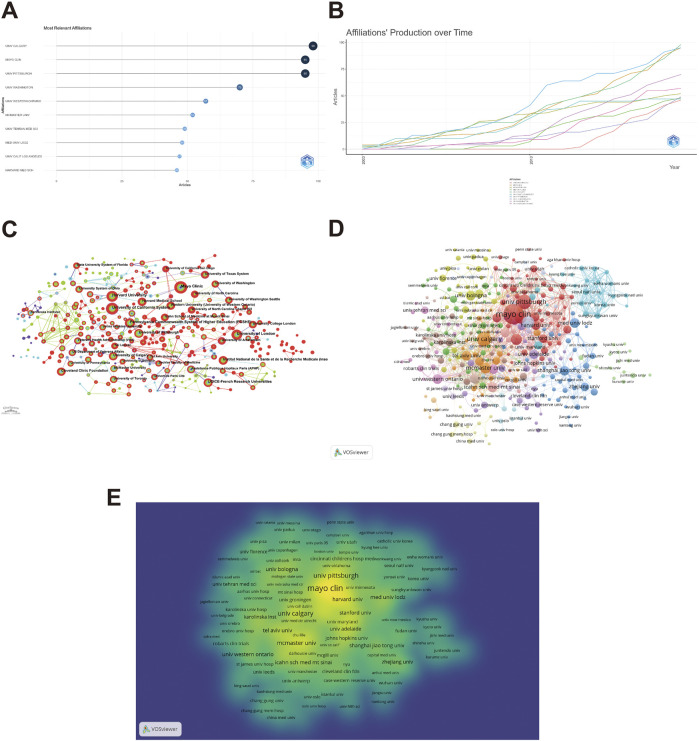
Visualization of institutions that performed research on abdominal pain in IBD in the past two decades. The number of publications by the Top 10 institutions **(A)**, the number of publications by the Top 10 institutions over time **(B)**, the co-occurrence of institutions **(C)**, the cooperation relationship between institutions **(D)** and the density visualization of cooperation relationship between institutions **(E)** in the field of abdominal pain in IBD in the past two decades.

The search results revealed that 11,208 research institutions have participated in abdominal pain-related investigations within the sphere of IBD research over the past 20 years. Notably, UNIV CALGARY emerged as the highest contributor with 98 publications, followed by Mayo Clin (95), Univ Pittsburgh (95), Univ Washington (70), And Univ Western Ontario (57) (Figure 5A). Furthermore, the bibliometric analysis demonstrated that the top 10 institutions in terms of publication output exhibited a marked upward trend in publication volume, underscoring the emergence of abdominal pain and IBD research as a nascent focal point (Figure 5B).

Utilizing CiteSpace, the collaborative networks of institutions engaged in abdominal pain-related research in the context of inflammatory bowel disease were examined, and a co-occurrence map was generated. Within the last two decades, 372 national/regional collaborations were published. Mayo Clinic was the leading institution in collaborative publications, followed by the University of California System, Harvard University, and the University of London (Figure 5C). VOSviewer was utilized to visually represent and analyze the graph of collaborations, specifically amongst institutions having published more than five papers. The collaboration network identified seventeen main clusters of authors. The red cluster boasts the most partnerships, including the Mayo Clinic, University of Pittsburgh, Harvard University, and Medical University Lodz. The green and blue clusters also exhibit collaborative relationships within and between clusters, forming a well-established research team (Figure 5D). Additionally, the Density Visualization diagram was utilized to depict the intensity and density of collaboration between various institutions. The color and size of each node correspond to the frequency and degree of cooperation, where a darker color indicates higher levels of collaboration, and a larger size indicates greater frequency. Amongst the institutions examined, the Mayo Clinic, the University of Pittsburgh, and the University of Calgary were the top three nodes in the density and intensity of cooperation metrics (Figure 5E).

### 3.5 Analysis of journals and literature citation

**FIGURE 6:**
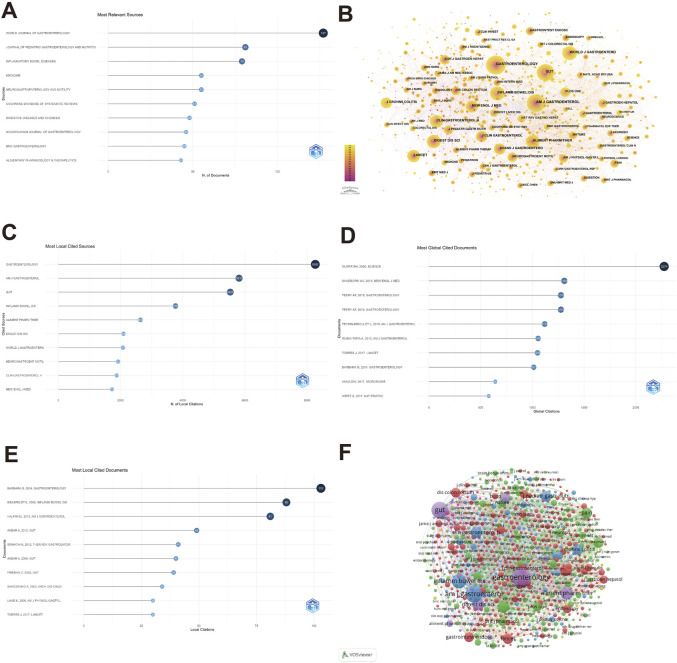
Visualization of journals and literature citation on abdominal pain in IBD in the past two decades. The number of publications by Top 10 journals **(A)**, the co-occurrence of cited journals **(B)**, the most locally cited journals **(C)**, the most globally cited documents **(D)**, the most locally cited documents **(E)** and the cooperation relationship between journals **(F)** in the field of abdominal pain in IBD in the past two decades.

Our search yielded numerous studies on abdominal pain in IBD research from 2003 to 2022, distributed across 990 journals. Table 2 highlights the ten most frequented journals about abdominal pain and IBD research, encompassing 630 publications (17.98%). Notably, the Cochrane Database of Systematic Reviews, with an impact factor (IF) of 11.874 and JCR classification of Q1, emerged as the journal with the highest IF. The World Journal of Gastroenterology (2021 IF = 5.374) made notable contributions to this area with 127 articles. In contrast, the Journal of Pediatric Gastroenterology And Nutrition (81), Inflammatory Bowel Diseases (79), Medicine (55), And Neurogastroenterology And Motility (55) followed suit (Figure 6A). It is worth mentioning that more than half of the journals boast Q1 or Q2 JCR rankings, which indicates that these journals maintain excellent publishing quality and standards regarding abdominal pain and IBD-related research. As such, researchers in related fields can confidently reference these journals as reliable and adequate resources (Table 2).

**TABLE 2:** The Top 10 journals with the most publications.

Top 10 journals with the most publications					
Rank	Journal	Publications	IF_2021_	JCR	SJR indicator 2021
1	WORLD JOURNAL OF GASTROENTEROLOGY	127	5.374	GASTROENTEROLOGY & HEPATOLOGY Q2	1.229
2	JOURNAL OF PEDIATRIC GASTROENTEROLOGY AND NUTRITION	81	3.355	GASTROENTEROLOGY & HEPATOLOGY Q3	0.919
3	INFLAMMATORY BOWEL DISEASES	79	7.29	GASTROENTEROLOGY & HEPATOLOGY Q1	1.706
4	MEDICINE	55	1.817	MEDICINE, GENERAL & INTERNAL Q3	0.47
5	NEUROGASTROENTEROLOGY AND MOTILITY	55	3.96	CLINICAL NEUROLOGY Q2 NEUROSCIENCES Q2	1.417
6	COCHRANE DATABASE OF SYSTEMATIC REVIEWS	51	11.874	MEDICINE, GENERAL & INTERNAL Q1	1.412
7	DIGESTIVE DISEASES AND SCIENCES	48	3.487	GASTROENTEROLOGY & HEPATOLOGY Q3	0.984
8	SCANDINAVIAN JOURNAL OF GASTROENTEROLOGY	46	3.027	GASTROENTEROLOGY & HEPATOLOGY Q4	0.651
9	BMC GASTROENTEROLOGY	45	2.848	GASTROENTEROLOGY & HEPATOLOGY Q4	0.737
10	ALIMENTARY PHARMACOLOGY & THERAPEUTICS	43	9.524	GASTROENTEROLOGY & HEPATOLOGY Q1	2.85

The phenomenon of co-citation refers to two journals being cited simultaneously in one or more publications, indicating an intellectual affinity between them. Utilizing CiteSpace analysis, the collaborative network of cited journals was investigated, and a co-occurrence visualization was obtained. Notably, Gastroenterology’s studies were cited the most (8,262), followed by AM J Gastroenterol (5,810), GUT (5,527), and Inflamm Bowel Dis (3,766) (Figures 6B,C). In the last two decades, DUERR’s article (Duerr et al., 2006) garnered the most citations on the Web of Science, with 2,274, and there were 60 studies with ≥200 citations (Figure 6D). In contrast, the article by BARBARA published in Gastroenterology (Barbara et al., 2004) received the most citations in the current study, with 103 (Figure 6E). Additionally, highly cited references were summarized (Supplementary Figure S3), along with the number of references per year (Supplementary Figure S4), with a particular reference (Barbara et al., 2004) obtaining the most citations.

Drawing on Bradford’s Law, we identified the core journals in the field of study, which can guide quality assessment and facilitate the selection of appropriate venues to advance academic research. Furthermore, this identification can streamline information search processes by prioritizing core journals in the field (Supplementary Figure S5). To visually and analytically evaluate the collaboration graph between journals with more than two publications, we employed VOSviewer. In the collaboration network, where five main clusters are evident, the red cluster boasts the most collaborators, including Gastrointest Endosc, Endoscopy, Lancet, and WORLD JOURNAL OF GASTROENTEROLOGY, followed by the green and blue clusters, which display intimate collaborative ties across clusters (Figure 6F).

### 3.6 Analysis of keywords and hotspots

#### 3.6.1 Words frequency analysis

**FIGURE 7:**
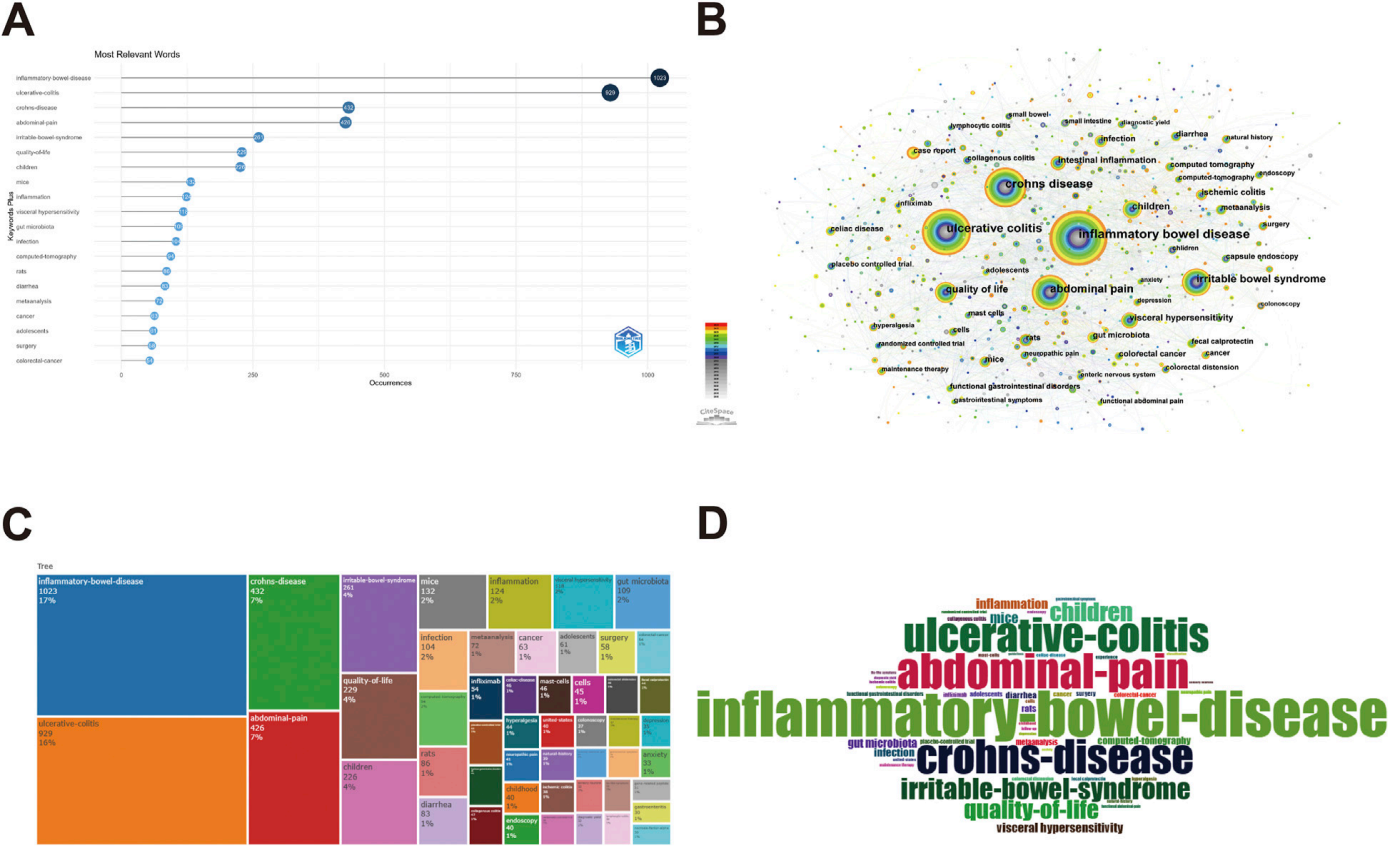
The keyword mapping of abdominal pain in IBD in the past two decades. The Top 10 relevant keywords **(A)**, the co-occurrence of relevant keywords **(B)**, the tree map of relevant keywords **(C)**, and the word cloud of relevant keywords **(D)** in the field of abdominal pain in IBD in the past two decades.

Keywords serve the purpose of compressing and summarizing the subject matter of scholarly literature. By tallying the occurrences of keywords in the research literature, it is possible to identify those that have received numerous citations, provided a partially indicative measure of their significance, and enabled an analysis of research trends (Liu et al., 2022). Bibliometric was used to analyze keywords, and in addition to search terms such as “ulcerative colitis”, “inflammatory bowel disease” and " abdominal pain ", this study also retrieved keywords for abdominal pain in IBD-related studies, such as “irritable bowel syndrome”, “quality of life”, “children”, “inflammation”, “visceral hypersensitivity”, “gut microbiota”, etc. (Figure 7A).

The analysis of keywords in IBD and abdominal pain was conducted using CiteSpace. Words such as “expression”, “gene expression”, and “RNA” were removed as they did not impact subsequent analysis. The keyword co-occurrence graph generated eight hundred forty-seven nodes and 3,033 links, each representing a keyword. The frequency of a keyword corresponds to its node size. The connections between nodes indicated their associations, with thicker connections signifying more significant co-occurrence and closeness. The color of the links indicated the time of keyword appearance, with cooler colors representing earlier appearances and warmer colors representing later ones. Of the 847 keywords obtained in the recent 20-year study, 112 had frequencies ≥20. Key influential nodes in the visualization included inflammatory bowel disease, ulcerative colitis, abdominal pain, Crohn’s disease, and irritable bowel syndrome. Notably, nodes such as “gut microbiota”, “case report”, “depression” and “maintenance therapy” primarily exhibited warm colors, indicating increased attention to these research topics in the field of abdominal pain and IBD-related research in recent years, culminating in emerging hotspots (Figure 7B). Additionally, Bibliometric analysis was employed to enhance keyword reliability and visualize them using word clouds and tree diagrams, revealing a trend in core keyword significance similar to the one previously discussed (Figures 7C,D).

#### 3.6.2 Cluster analysis

**FIGURE 8:**
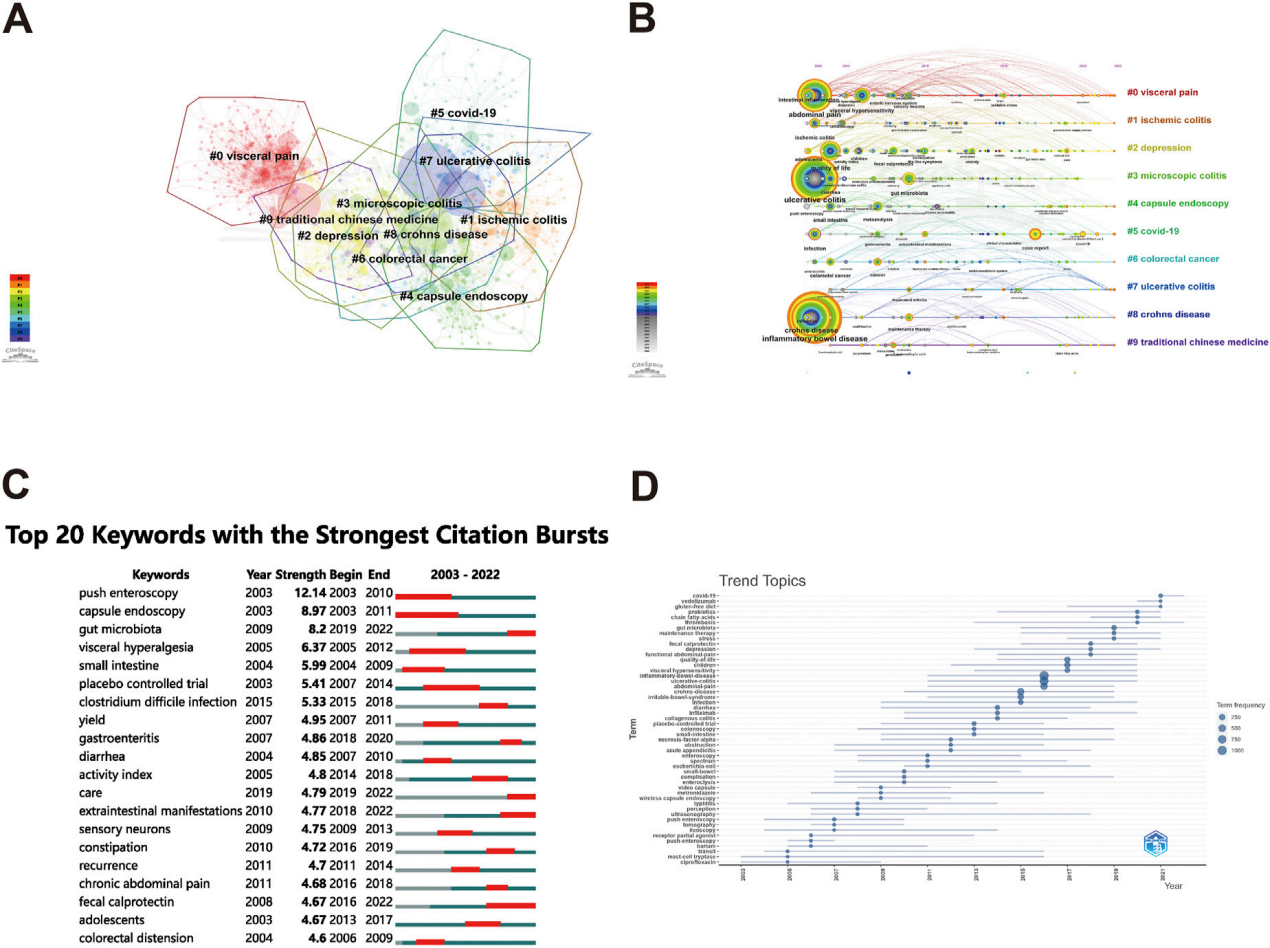
Visualization analysis of keywords cluster and research hotspots and evolution trends on abdominal pain in IBD in the past two decades. The clustering map of relevant keywords **(A)**, the clustering timeline view of relevant keywords **(B)**, the Top 20 keywords with the strongest citation bursts **(C)**, and the trend topics of relevant keywords **(D)** in the field of abdominal pain in IBD in the past two decades.

The operation of keyword clustering involves identifying related keywords that commonly appear in research articles and grouping closely related words into one category to extract concealed information. The present study utilized CiteSpace to cluster and analyze the main keywords, categorizing the clusters using the log-likelihood ratio (LLR) method to generate both a clustering view and a timeline view (Figures 8A,B). The clustering map of keywords provided an overview of the research interests in the field. Over the last two decades, ten meaningful clusters were formed, numbered from 0 to 9, with lower numbers indicating greater keyword inclusion within clusters. The keyword clustering graph revealed multiple overlapping clusters, indicating their close association. Using identical colors for clusters facilitated clear representation and strengthened correlations between clusters, collectively pointing towards similar research areas. The timeline view of the clustering graph visualizes the period of each cluster formation and the linkage between them, depicting the evolution of research topics. The X-axis of the timeline view represented the year of publication, while the Y-axis corresponded to the cluster number. The resultant cluster analysis identified areas of research interest that included visceral pain, depression, Covid-19, ischemic colitis, capsule endoscopy, and traditional Chinese medicine (Table 3).

**TABLE 3:** The clusters in the field of abdominal pain in IBD.

ID	Cluster	Size	Silhouette	Year
0	visceral pain	166	0.77	2010
1	ischemic colitis	113	0.744	2011
2	depression	112	0.74	2011
3	microscopic colitis	88	0.768	2010
4	capsule endoscopy	77	0.78	2012
5	covid-19	69	0.834	2011
6	colorectal cancer	51	0.728	2010
7	ulcerative colitis	47	0.841	2015
8	Crohn’s disease	45	0.821	2010
9	traditional Chinese medicine	34	0.786	2014

#### 3.6.3 Research hotspots and evolution trends

Kleinberg’s burst-detection algorithm is an original computational method that identifies sharp changes in events, which can be utilized to detect sudden increases in research interest in a particular field. A sudden surge in the frequency of keywords could signify a key direction of development trends (Kleinberg, 2003). The present study employed CiteSpace software to analyze the keywords about abdominal pain in IBD-related research from 2003 to 2022, mapping the evolution of emerging high-intensity keywords to gain insights into current research hotspots and trends. Figure 8C presents the top 20 keywords ranked by emergent intensity, with the duration of the emergent keywords being indicated by the red line. The keyword “push enteroscopy” topped the list with a strength of 12.14, followed by capsule endoscopy (8.97), gut microbiota (8.2), visceral hyperalgesia (6.37), and fecal calprotectin (4.67). Furthermore, the emergent keywords that persisted through to 2022 included “gut microbiota”, “extraintestinal manifestations”, “care”, and “fecal calprotectin”, among others, indicating that these topics represent hotspots within the field of interest (Figure 8C). To clearly illustrate the trend of these hotspots over time, we analyzed the keywords in each period, providing scholars engaged in abdominal pain in IBD-related research with scientific guidance and potential future research directions (Figure 8D). The comprehensive analysis of keywords and research hotspots suggests that gut microbiota, depression, stress, fecal calprotectin, and multidisciplinary approach may become dominant research foci in IBD research regarding abdominal pain.

## 4 Discussion

### 4.1 General information

Abdominal pain can harm different areas of daily life, particularly affecting the quality of life of people with IBD and increasing the psychosocial burden (Wils et al., 2022). With the burgeoning field of pain research, abdominal pain is increasingly being investigated in the field of IBD. Therefore, this study provides a systematic analysis of abdominal pain topics in inflammatory bowel disease research using a bibliometric approach to understand better the current state of research and future research trends in the field.

In this study, we analyzed IBD and abdominal pain documents and reviewed the research results and progress using CiteSpace and VOSviewer quantitative analysis software. The basic information of annual publication quantity, country, author, institution, discipline, and journal is quantitatively analyzed. Through the search of the scholarly literature concerning abdominal pain in inflammatory bowel disease from 2003 to 2022, our analysis discerned an ongoing year-on-year escalation in the total volume of published research articles in this domain. Such a trend suggests a growing interest and potential for advancement in this field among scholars.

Based on the statistical analysis of the number of papers published by various countries/regions and institutions, it is possible to identify key countries/regions and research institutions that have published many IBD and abdominal pain documents and have greater influence and determine their cooperative relationship. United States, China, and JAPAN are the countries that mainly study IBD and abdominal pain. Research institutions from the United States currently dominate abdominal pain research in inflammatory bowel disease, with half of the top 10 institutions in terms of literature published Mayo Clin, Univ Pittsburgh, And Univ Washington, among others. Meanwhile, institutions in Canada are not far behind. Furthermore, the cooperation between various countries and institutions is relatively close. Close cooperation and communication between countries and institutions are conducive to eliminating academic barriers and further developing IBD and abdominal pain related research. The strongest collaboration is between the United States and Canada, and the United States has published the most articles on its collaboration. In particular, the United States is more frequent in promoting international cooperation.

From the perspective of the author, the most published author in this paper is Feagan, Brian G. from Alimentiv Inc, Canada, who surprisingly performs well in terms of the number of publications and the quality of publications (H-index = 19). Dr. Brian Feagan is a gastroenterologist. His research focus is the design, conduct and execution of large-scale randomized controlled trials (RCTs) in CD and UC, and over the past 30 years, has been Principal Investigator in over 140 multi-center RCTs. His research has been devoted to the development, validation and optimization of outcome measures to assess the efficacy of novel therapeutics in CD and UC. For example, an article published in the journal Inflammatory Bowel Diseases describes the correlation of stool frequency and abdominal pain measurements with simple endoscopy score for the CD in 2022 (Lewis et al., 2020). Also, Sandborn, William J from the United States the author with the highest average citation rate, indicating that they play a pivotal role in this field of research. William Sandborn, MD, is a board-certified gastroenterologist who is one of the world’s top experts in the management of UC and CD. His clinical trials have been instrumental to developing modern treatments for IBD. The professor’s randomized controlled trial study, published in 2022, examined the association between patient-reported general wellbeing relative to symptoms of diarrhea and abdominal pain in patients with moderate to severe CD was explored (Sandborn et al., 2022).

As prestigious scientific journals are highly coveted in the academic world, the impact factor (IF) of a given journal is a powerful indicator of citation performance to a certain extent. Typically, top-tier journals tend to publish articles that attract high citation rates. Accordingly, this research suggests that articles in this field are largely published in journals such as World Journal Of Gastroenterology and Inflammatory Bowel Diseases, which boast IFs of 5.37and 7.29, respectively. Moreover, ranking the top 10 journals in terms of published literature reveals that half of them are categorized as Q1 or Q2 in the JCR division, indicating that the quality standards for publication in abdominal pain and IBD-related research journals are relatively high. Thus, the research area holds significant value in the global research domain.

### 4.2 Hotspots and frontiers

Analysis of high-frequency keywords reflects the hotspots in a particular research field. We used key co-occurrence analysis to determine the main directions and hotspots in IBD and abdominal pain, as well as to uncover the development and changes of its theme structure. Using CiteSpace for visual analysis of keywords, the research area was found to be associated with buzzwords such as “irritable bowel syndrome”, “quality of life”, “children”, “inflammation”, “visceral hypersensitivity”, “gut microbiota”, “fecal calprotectin”, “case report”, “depression” and “multidisciplinary approach” among others. Emergent keywords and the thematic evolution of keywords attest to this observation.

#### 4.2.1 Mechanisms of abdominal pain generation

As the research continues and expands, from the clustering and co-occurrence analysis of keywords, we find that the research focuses more on inflammation, irritable bowel syndrome, visceral hypersensitivity, gut microbiota, integrated management of pain, and psychosocial factors, including depression and anxiety. The onset of abdominal pain can typically be attributed to the initial activation of visceral sensory neurons (nociceptive receptors), which serve as the conduits for nociceptive information transmission to the central nervous system (CNS) by way of the dorsal horn located in the spinal cord. This information ultimately integrates within the brain to elicit unpleasant sensory and emotional experiences (Hurtado-Lorenzo et al., 2021). Neuroplasticity drives the sensitization of visceral afferent fibers, resulting in a perceptual shift toward sensory stimuli. Injury-related receptor sensitization typically occurs in response to tissue damage or inflammation. It is characterized by lowered thresholds and heightened responsiveness to noxious stimuli or visceral hypersensitivity (Enck et al., 2016). As a primary mechanism for abdominal pain, significant alterations within the brain-gut axis are involved, consisting of a complex network of afferent and efferent neural pathways that link cognitive, emotional, and autonomic brain centers with neuroendocrine centers, the enteric nervous system, the gut microbiota, and the immune system (Regueiro et al., 2017).

The production of abdominal pain involves multiple mechanisms, including inflammatory immune activation and neuroplasticity, with inflammatory mediators playing a significant role in altering the sensitivity of nociceptive receptors. During active IBD, pro-inflammatory mediators are released and interact with receptors to cause pain, including adenosine triphosphate, histamine, IL-1β, proteases, and bradykinin (Lakhan and Kirchgessner, 2010). Other inflammatory mediators, like IL-6 and TNF-α, induce nociceptive receptor sensitization indirectly. Nociceptors contribute to local neuroinflammation by generating inflammatory mediators via axonal reflexes, such as substance P, neurokinin A, ATP, and calcitonin gene-related peptides. Even when IBD is resting, mast cells may stimulate visceral neuronal signaling, as a study by van Hoboken et al. shows (Bielefeldt et al., 2009). The study highlights the role of mast cells in mediating visceral hypersensitivity responses in patients with IBD, as well as the significant correlation between mast cell density and pain perception in patients with UC in remission. Despite these findings, the mediators contributing to post-inflammatory changes in the enteric nervous system leading to visceral pain are yet to be fully elucidated (Lakhan and Kirchgessner, 2010; van Hoboken et al., 2011).

#### 4.2.2 Mechanisms of peripheral sensitization and central hypersensitivity in abdominal pain associated with IBD

Numerous reports have described potential molecular mechanisms underlying peripheral sensitization observed in animal models of colitis and patients with IBD. Notably, significant upregulation of Transient Receptor Potential Cation Channel Subfamily V Member 1 (TRPV1) expression has been detected in colonic tissues of both UC animals and patients. It remains elevated even in clinical remission (Akbar et al., 2010; Lapointe et al., 2015; Duo et al., 2020). TRPV1 is a non-selective cation channel that transmits pain and visceral sensation and is commonly known as a “heat receptor”. Transient Receptor Potential Cation Channel Subfamily M Member 8 (TRPM8), another member of the transient receptor ion channel protein (TRP) family, is activated by cold stimuli (<26°C) and chemicals eliciting a cold sensation (Bautista et al., 2007; Alaimo and Rubert, 2019). It is regarded as a “cold receptor”, with acute TRPM8 activation shown to alleviate neuropathic and visceral pain, hinting at a possible neurogenic anti-inflammatory effect in specific contexts (Proudfoot et al., 2006; Brignell et al., 2008; Harrington et al., 2011). Activating TRPV1 transduces and relays noxious stimuli and triggers neuropeptide release, including substance P and calcitonin gene-related peptide (CGRP), from sensory neuron perineuronal nerve endings, resulting in colonic neurogenic inflammation (Averbeck and Reeh, 2001; Farmer and Aziz, 2013). Additionally, pro-inflammatory mediators of mast cells, such as histamine, have been shown to mediate the sensitization effect of TRPV1 (Perna et al., 2021). Collectively, these research findings suggest that sensory nerve function alterations and visceral hypersensitivity formation in the presence of inflammation and post-inflammation play essential roles in painful symptoms’ persistence and mucosal inflammation development in IBD.

The activation of Transient Receptor Potential Cation Channel Subfamily V Member 4 (TRPV4) within the gastrointestinal tract has been linked to increased epithelial barrier permeability, chemokine secretion, and immune cell recruitment (Rizopoulos et al., 2018; Chen et al., 2020). Notably, TRPV4 mRNA expression and TRPV4 immunoreactivity were notably significantly higher in colonic biopsies obtained from IBD patients than in healthy controls (Fichna et al., 2012). The administration of TRPV4-selective and non-selective antagonists in a mouse model of colitis significantly reduced macroscopic damage, myeloperoxidase activity, and pain reduction; conversely, TRPV4 agonist treatment yielded opposite results (D’Aldebert et al., 2011). Specifically, in a Dextran Sulfate Sodium (DSS) induced colitis model, TRPV4 knockout mice displayed significantly reduced intestinal inflammation compared to their wild-type counterparts (Matsumoto et al., 2018). These findings suggest that TRPV4 activation serves pro-hypersensitivity and pro-inflammatory functions during colitis and that TRPV4 antagonists exhibit potential as effective therapeutic targets in animal models of colitis.

Abdominal pain models in IBD suggest sensitization occurs due to ongoing intestinal inflammation. Peripheral sensitization is widely believed to be the fundamental driver of acute onset pain in IBD, while central hypersensitivity is vital to chronic pain manifestation following inflammation (Farrell et al., 2014). During inflammatory states such as colitis, ion channels accountable for mechanotransduction and pain-related action potential generation are upregulated. Such ion channels include NMDA and AMPA receptors, along with mGLUR receptors, with glutamate able to cause sustained neuroexcitability, even without inflammatory stimuli, when activating the previously mentioned receptors (Zhou and Nicholas, 2008; Vermeulen et al., 2014). Additionally, activated microglia residing in the dorsal horn of the spinal cord, particularly in superficial layers I-II, primarily respond to injury or inflammation-related nociceptive input. In animal models of neuropathic pain, spinal cord astrocytes are activated following inflammation, and inhibiting astrocyte activation reduces inflammatory hyperalgesia (Morales-Soto and Gulbransen, 2019). Activated glial cells produce several pro-inflammatory mediators, such as TNF-α, which contribute to sensitizing dorsal horn nerve fibers (Ji et al., 2013).

#### 4.2.3 Stress

Stress pertains to the response of an organism to deviations from its homeostatic conditions and plays a significant role in the intricate interplay between the brain and gut. Psychological stress triggers the activation of the hypothalamic-pituitary-adrenal (HPA) axis, inducing the release of corticotropin-releasing factor (CRF) from the paraventricular nucleus of the hypothalamus. This, in turn, facilitates the discharge of adrenocorticotropic hormones from the pituitary into the systemic circulation, culminating in the synthesis and secretion of cortisol from the adrenal cortex (Chrousos, 2009; Boeckxstaens et al., 2016). CRF-R1 and CRF-R2 selectively target the enteric nervous system, enterochromaffin (EC), and immune cells (Greenwood-Van et al., 2016; Tache et al., 2018). Evidence indicates that CRF promotes the degranulation of mast cells in experimental animals and humans’ colon through CRF-R1, leading to the generation of various mediators, including histamine, 5-HT, tryptophanase, prostaglandin E2, and nerve growth factor (Wallon et al., 2008). These mediators can perturb intestinal barrier function, resulting in augmented digestive tract permeability and activation of spinal sensory afferents, ultimately leading to visceral hypersensitivity. Conversely, CRF-R2 is believed to curb the action of CRF-R1 by modulating neurotransmitter release from enteric muscle neurons (Barbara et al., 2007; Larauche et al., 2008; Wu et al., 2011).

#### 4.2.4 Gut microbiota

The gut microbiota has been implicated in modulating visceral pain sensitivity. Signaling molecules from the gut microbes, such as microbial by-products, metabolites, transmitters, and modulators, may influence neuroinflammation processes, resulting in peripheral and central sensitization (Guo et al., 2019). Although the etiology is obscure, the increased number of harmful species or the depletion of beneficial bacteria due to prolonged antibiotic use has been associated with visceral hypersensitivity. For instance, administering antibiotics effectively reduced visceral pain in murine models induced by intraperitoneal acetic acid or capsaicin applied to the colon (Aguilera et al., 2015). Conversely, the application of certain *Lactobacillus* strains augmented the expression of cannabinoid receptors in the intestinal epithelial cells, conveying analgesic functions within the digestive tract in a mouse model (Rousseaux et al., 2007). Likewise, clinical trials have successfully demonstrated the effectiveness of certain probiotics in relieving functional abdominal pain; these probiotics include *Lactobacillus* rhamnosus GG, a mixture of Bifidobacterium infantis M-63, breve M-16V, and longum BB536, as well as *Lactobacillus* acidophilus NCFM (Rousseaux et al., 2007; Horvath et al., 2011; Ringel-Kulka et al., 2014). Notably, fecal microbiota transplantation was employed to establish that visceral allergy in rats could be caused by engraftment with gut microbiota obtained from IBS patients afflicted by visceral allergy (Saulnier et al., 2011). Taken together, emerging findings underscore the likelihood of a correlation between gut microbiota and visceral pain, given that changes in the gut microbiota are closely linked to the pathogenesis of IBD and post-inflammatory pain in IBD patients (van Thiel et al., 2020).

#### 4.2.5 Psychological factors and quality of life

Post-inflammatory pain resulting from IBD significantly impacts the quality of life of patients. Pain in adult and pediatric patients has been associated with several psychological factors, including depression, anxiety, and stress (Neuendorf et al., 2016). Some studies have demonstrated that a greater number of patients with IBD suffer from anxiety and depression compared to the general population. Research conducted on hospitalized patients displayed that IBD patients afflicted with chronic pain were likelier to exhibit lower mental health as evidenced by elevated anxiety and depression scores (Morrison et al., 2013). Distinctly, patients in remission from UC experiencing IBS-like symptoms, such as abdominal pain, demonstrated higher levels of anxiety, depression, and stress than those experiencing remission without IBS-like symptoms (Jonefjall et al., 2016). Pain in individuals often elicited an emotional response, consequently hypothesized to cause hypervigilance in recurring episodes. It has been hypothesized that individuals with a proclivity toward pain disorders may also be prone to developing psychological disorders, such as anxiety and depression. Individuals with psychological disorders were also postulated to activate their pain and stress circuits more frequently, contributing to their sensitivity (Sweeney et al., 2020).

#### 4.2.6 Social factors

Pain resulting from IBD can coincide with several social factors, including employment status, social deprivation, social attachment styles, and early childhood adversity (Caplan et al., 2014). Research conducted on Korean patients with inactive IBD indicated that pain was linked to socioeconomic deprivation. Early childhood is critical for developing brain circuits that regulate stress and pain perception (Kim et al., 2016). Different factors may modify how these circuits develop in early life, mainly if a genetic predisposition to hypersensitivity exists. Factors such as mental/emotional trauma, major illness, infection, or childhood injury may be included in this category (Takahashi et al., 2021). Additionally, it is believed that chronic pain exhibits a significant genetic component, with specific individuals genetically predisposed to developing hypersensitivity reactions biased toward enhanced pain perception (Zorina-Lichtenwalter et al., 2016). Recently, the Swiss IBD Cohort Study identified two significant SNPs, 1042713 (located on the ADRB2 gene) and rs4663866 (near the HES6 gene), that were associated with IBS and levels of abdominal pain in patients with UC (Ledergerber et al., 2021).

### 4.3 Advantages and limitations

This study has several unique advantages. Firstly, we systematically analyzed research of abdominal pain in IBD by bibliometrics for the first time, which can provide comprehensive guidance for scholars who pay attention to related research. Secondly, we used four bibliometric tools simultaneously for the survey, four of which (R, VOSviewer, CiteSpace and bibliometric online analysis platform) have been widely used in the field of bibliometrics, so our data analysis process is very likely to be objective. Finally, bibliometric analysis provides more complete insight into the hotspots and frontiers than traditional reviews.

Several limitations are inherent to this study. Firstly, this study solely analyzed English language publications, potentially disregarding valuable literature from non-English sources. Additionally, the limitation of software-based scientific measurement added the difficulty in combining at least two databases for the analysis. The study exclusively relied on the Web of Science literature without incorporating other databases, such as PubMed and Scopus. Some influential publications were excluded and could have marginally impacted the findings. Therefore, more databases should be employed for a more rigorous analysis in the future.

## 5 Conclusion

Considerable research has been focused on the etiology and treatment of abdominal pain in IBD; nevertheless, numerous uncertainties remain in this sphere of inquiry. Through bibliometric and visual analyses, this study aims to elucidate the intricate regulatory networks involved in abdominal pain associated with IBD, while also providing insight into the contemporary state of progress and areas of concern in this field. Specifically, it has been established that abdominal pain in IBD is intricately linked to both peripheral and central sensitization processes and various psychophysiological factors. To gain a more comprehensive understanding of the multifaceted mechanisms underlying abdominal pain in IBD, a thorough exploration of the interactions among multiple causative factors, including clinical psychophysiological phenotypes, molecular mechanisms, and multi-omics data, is imperative. Presently available treatment options for this malady are limited, and new therapies consistent with underlying pathophysiology are required to enhance clinical outcomes. Moreover, altered interactions between the brain and gut are embedded in IBD, and psychological and social factors are recognized to impair patient quality of life and impact the course of the disease. Future trends in the management of abdominal pain necessitate the integration of multidisciplinary teams and precision treatment.

## Data Availability

The original contributions presented in the study are included in the article/Supplementary Material, further inquiries can be directed to the corresponding author.

## References

[B1] Aguilera M. Cerda-Cuellar M. Martinez V. (2015). Antibiotic-induced dysbiosis alters host-bacterial interactions and leads to colonic sensory and motor changes in mice. Gut Microbes 6, 10–23. 10.4161/19490976.2014.990790 25531553 PMC4615720

[B2] Akbar A. Yiangou Y. Facer P. Brydon W. G. Walters J. R. Anand P. (2010). Expression of the TRPV1 receptor differs in quiescent inflammatory bowel disease with or without abdominal pain. Gut 59, 767–774. 10.1136/gut.2009.194449 20551462

[B3] Alaimo A. Rubert J. (2019). The pivotal role of TRP channels in homeostasis and diseases throughout the gastrointestinal tract. Int. J. Mol. Sci. 20, 5277. 10.3390/ijms20215277 31652951 PMC6862298

[B4] Averbeck B. Reeh P. W. (2001). Interactions of inflammatory mediators stimulating release of calcitonin gene-related peptide, substance P and prostaglandin E(2) from isolated rat skin. Neuropharmacology 40, 416–423. 10.1016/s0028-3908(00)00171-4 11166334

[B5] Bakshi N. Hart A. L. Lee M. C. Williams A. Lackner J. M. Norton C. (2021). Chronic pain in patients with inflammatory bowel disease. Pain 162, 2466–2471. 10.1097/j.pain.0000000000002304 34534174 PMC8442739

[B6] Barbara G. Stanghellini V. De Giorgio R. Cremon C. Cottrell G. S. Santini D. (2004). Activated mast cells in proximity to colonic nerves correlate with abdominal pain in irritable bowel syndrome. Gastroenterology 126, 693–702. 10.1053/j.gastro.2003.11.055 14988823

[B7] Barbara G. Wang B. Stanghellini V. de Giorgio R. Cremon C. Di Nardo G. (2007). Mast cell-dependent excitation of visceral-nociceptive sensory neurons in irritable bowel syndrome. Gastroenterology 132, 26–37. 10.1053/j.gastro.2006.11.039 17241857

[B8] Bautista D. M. Siemens J. Glazer J. M. Tsuruda P. R. Basbaum A. I. Stucky C. L. (2007). The menthol receptor TRPM8 is the principal detector of environmental cold. Nature 448, 204–208. 10.1038/nature05910 17538622

[B9] Bielefeldt K. Davis B. Binion D. G. (2009). Pain and inflammatory bowel disease. Inflamm. Bowel Dis. 15, 778–788. 10.1002/ibd.20848 19130619 PMC3180862

[B10] Bisgaard T. H. Allin K. H. Keefer L. Ananthakrishnan A. N. Jess T. (2022). Depression and anxiety in inflammatory bowel disease: epidemiology, mechanisms and treatment. Nat. Rev. Gastroenterol. Hepatol. 19, 717–726. 10.1038/s41575-022-00634-6 35732730

[B11] Boeckxstaens G. Camilleri M. Sifrim D. Houghton L. A. Elsenbruch S. Lindberg G. (2016). Fundamentals of neurogastroenterology: physiology/motility - sensation. Gastroenterology 150, 1292–1304.e2. 10.1053/j.gastro.2016.02.030 27144619

[B12] Brignell J. L. Chapman V. Kendall D. A. (2008). Comparison of icilin- and cold-evoked responses of spinal neurones, and their modulation of mechanical activity, in a model of neuropathic pain. Brain Res. 1215, 87–96. 10.1016/j.brainres.2008.03.072 18479674

[B13] Caplan R. A. Maunder R. G. Stempak J. M. Silverberg M. S. Hart T. L. (2014). Attachment, childhood abuse, and IBD-related quality of life and disease activity outcomes. Inflamm. Bowel Dis. 20, 909–915. 10.1097/MIB.0000000000000015 24651585

[B14] Chen Y. Mu J. Zhu M. Mukherjee A. Zhang H. (2020). Transient receptor potential channels and inflammatory bowel disease. Front. Immunol. 11, 180. 10.3389/fimmu.2020.00180 32153564 PMC7044176

[B15] Chrousos G. P. (2009). Stress and disorders of the stress system. Nat. Rev. Endocrinol. 5, 374–381. 10.1038/nrendo.2009.106 19488073

[B16] D'Aldebert E. Cenac N. Rousset P. Martin L. Rolland C. Chapman K. (2011). Transient receptor potential vanilloid 4 activated inflammatory signals by intestinal epithelial cells and colitis in mice. Gastroenterology 140, 275–285. 10.1053/j.gastro.2010.09.045 20888819

[B17] Deng P. Wang S. Sun X. Qi Y. Ma Z. Pan X. (2022). Global trends in research of gouty arthritis over past decade: A bibliometric analysis. Front. Immunol. 13, 910400. 10.3389/fimmu.2022.910400 35757713 PMC9229989

[B18] Duerr R. H. Taylor K. D. Brant S. R. Rioux J. D. Silverberg M. S. Daly M. J. (2006). A genome-wide association study identifies IL23R as an inflammatory bowel disease gene. Science 314, 1461–1463. 10.1126/science.1135245 17068223 PMC4410764

[B19] Duo L. Wu T. Ke Z. Hu L. Wang C. Teng G. (2020). Gain of function of ion channel TRPV1 exacerbates experimental colitis by promoting dendritic cell activation. Mol. Ther. Nucleic Acids 22, 924–936. 10.1016/j.omtn.2020.10.006 33251043 PMC7666365

[B20] Enck P. Aziz Q. Barbara G. Farmer A. D. Fukudo S. Mayer E. A. (2016). Irritable bowel syndrome. Nat. Rev. Dis. Prim. 2, 16014. 10.1038/nrdp.2016.14 27159638 PMC5001845

[B21] Farmer A. D. Aziz Q. (2013). Gut pain & visceral hypersensitivity. Br. J. Pain 7, 39–47. 10.1177/2049463713479229 26516496 PMC4590155

[B22] Farrell K. E. Callister R. J. Keely S. (2014). Understanding and targeting centrally mediated visceral pain in inflammatory bowel disease. Front. Pharmacol. 5, 27. 10.3389/fphar.2014.00027 24634658 PMC3942649

[B23] Fichna J. Mokrowiecka A. Cygankiewicz A. I. Zakrzewski P. K. Malecka-Panas E. Janecka A. (2012). Transient receptor potential vanilloid 4 blockade protects against experimental colitis in mice: a new strategy for inflammatory bowel diseases treatment? Neurogastroenterol. Motil. 24, e557–e560. 10.1111/j.1365-2982.2012.01999.x 22882778

[B24] Greenwood-Van M. B. Moloney R. D. Johnson A. C. Vicario M. (2016). Mechanisms of stress-induced visceral pain: implications in irritable bowel syndrome. J. Neuroendocrinol. 28, 12361. 10.1111/jne.12361 26749172

[B25] Guan L. Liu Y. Wu B. Chen A. Tao W. Lin C. (2022). Research hotspots and trends in visceral pain research: A global comprehensive bibliometric analysis. Front. Mol. Neurosci. 15, 1022463. 10.3389/fnmol.2022.1022463 36683850 PMC9848657

[B26] Guo R. Chen L. H. Xing C. Liu T. (2019). Pain regulation by gut microbiota: molecular mechanisms and therapeutic potential. Br. J. Anaesth. 123, 637–654. 10.1016/j.bja.2019.07.026 31551115

[B27] Harrington A. M. Hughes P. A. Martin C. M. Yang J. Castro J. Isaacs N. J. (2011). A novel role for TRPM8 in visceral afferent function. Pain 152, 1459–1468. 10.1016/j.pain.2011.01.027 21489690

[B28] Horvath A. Dziechciarz P. Szajewska H. (2011). Meta-analysis: lactobacillus rhamnosus GG for abdominal pain-related functional gastrointestinal disorders in childhood. Aliment. Pharmacol. Ther. 33, 1302–1310. 10.1111/j.1365-2036.2011.04665.x 21507030

[B29] Hurtado-Lorenzo A. Honig G. Weaver S. A. Larkin P. B. Heller C. (2021). Chronic abdominal pain in IBD research initiative: unraveling biological mechanisms and patient heterogeneity to personalize treatment and improve clinical outcomes. Crohns Colitis 360, otab034. 10.1093/crocol/otab034 PMC9802354 36776666

[B30] Ji R. R. Berta T. Nedergaard M. (2013). Glia and pain: is chronic pain a gliopathy? Pain 154 (1), S10–S28. 10.1016/j.pain.2013.06.022 23792284 PMC3858488

[B31] Jonefjall B. Ohman L. Simren M. Strid H. (2016). IBS-like symptoms in patients with ulcerative colitis in deep remission are associated with increased levels of serum cytokines and poor psychological well-being. Inflamm. Bowel Dis. 22, 2630–2640. 10.1097/MIB.0000000000000921 27636379

[B32] Kim M. C. Jung Y. S. Song Y. S. Lee J. I. Park J. H. Sohn C. I. (2016). Factors associated with anxiety and depression in Korean patients with inactive inflammatory bowel disease. Gut Liver 10, 399–405. 10.5009/gnl15188 26470768 PMC4849693

[B33] Kleinberg J. (2003). Bursty and hierarchical structure in streams. Data Min. Knowl. Discov. 7, 373–397. 10.1023/A:1024940629314

[B34] Lakhan S. E. Kirchgessner A. (2010). Neuroinflammation in inflammatory bowel disease. J. Neuroinflammation 7, 37. 10.1186/1742-2094-7-37 20615234 PMC2909178

[B35] Lapointe T. K. Basso L. Iftinca M. C. Flynn R. Chapman K. Dietrich G. (2015). TRPV1 sensitization mediates postinflammatory visceral pain following acute colitis. Am. J. Physiol. Gastrointest. Liver Physiol. 309, G87–G99. 10.1152/ajpgi.00421.2014 26021808

[B36] Larauche M. Bradesi S. Million M. McLean P. Tache Y. Mayer E. A. (2008). Corticotropin-releasing factor type 1 receptors mediate the visceral hyperalgesia induced by repeated psychological stress in rats. Am. J. Physiol. Gastrointest. Liver Physiol. 294, G1033–G1040. 10.1152/ajpgi.00507.2007 18308857

[B37] Ledergerber M. Lang B. M. Heinrich H. Biedermann L. Begre S. Zeitz J. (2021). Abdominal pain in patients with inflammatory bowel disease: association with single-nucleotide polymorphisms prevalent in irritable bowel syndrome and clinical management. Bmc Gastroenterol. 21, 53. 10.1186/s12876-021-01622-x 33546600 PMC7866750

[B38] Lewis J. D. Rutgeerts P. Feagan B. G. D'Haens G. Danese S. Colombel J. F. (2020). Correlation of stool frequency and abdominal pain measures with simple endoscopic score for Crohn's disease. Inflamm. Bowel Dis. 26, 304–313. 10.1093/ibd/izz241 31644790

[B39] Li K. Rollins J. Yan E. (2018). Web of science use in published research and review papers 1997-2017: a selective, dynamic, cross-domain, content-based analysis. Scientometrics 115, 1–20. 10.1007/s11192-017-2622-5 29527070 PMC5838136

[B40] Liu C. Yu R. Zhang J. Wei S. Xue F. Guo Y. (2022). Research hotspot and trend analysis in the diagnosis of inflammatory bowel disease: A machine learning bibliometric analysis from 2012 to 2021. Front. Immunol. 13, 972079. 10.3389/fimmu.2022.972079 36189197 PMC9516000

[B41] Matsumoto K. Yamaba R. Inoue K. Utsumi D. Tsukahara T. Amagase K. (2018). Transient receptor potential vanilloid 4 channel regulates vascular endothelial permeability during colonic inflammation in dextran sulphate sodium-induced murine colitis. Br. J. Pharmacol. 175, 84–99. 10.1111/bph.14072 29053877 PMC5740260

[B42] Morales-Soto W. Gulbransen B. D. (2019). Enteric glia: A new player in abdominal pain. Cell Mol. Gastroenterol. Hepatol. 7, 433–445. 10.1016/j.jcmgh.2018.11.005 30739868 PMC6369218

[B43] Morrison G. Van Langenberg D. R. Gibson S. J. Gibson P. R. (2013). Chronic pain in inflammatory bowel disease: characteristics and associations of a hospital-based cohort. Inflamm. Bowel Dis. 19, 1210–1217. 10.1097/MIB.0b013e318280e729 23524595

[B44] Neuendorf R. Harding A. Stello N. Hanes D. Wahbeh H. (2016). Depression and anxiety in patients with inflammatory bowel disease: A systematic review. J. Psychosom. Res. 87, 70–80. 10.1016/j.jpsychores.2016.06.001 27411754

[B45] Norton C. Czuber-Dochan W. Artom M. Sweeney L. Hart A. (2017). Systematic review: interventions for abdominal pain management in inflammatory bowel disease. Aliment. Pharmacol. Ther. 46, 115–125. 10.1111/apt.14108 28470846

[B46] Peng C. Kuang L. Zhao J. Ross A. E. Wang Z. Ciolino J. B. (2022). Bibliometric and visualized analysis of ocular drug delivery from 2001 to 2020. J. Control Release 345, 625–645. 10.1016/j.jconrel.2022.03.031 35321827

[B47] Perna E. Aguilera-Lizarraga J. Florens M. V. Jain P. Theofanous S. A. Hanning N. (2021). Effect of resolvins on sensitisation of TRPV1 and visceral hypersensitivity in IBS. Gut 70, 1275–1286. 10.1136/gutjnl-2020-321530 33023902

[B48] Proudfoot C. J. Garry E. M. Cottrell D. F. Rosie R. Anderson H. Robertson D. C. (2006). Analgesia mediated by the TRPM8 cold receptor in chronic neuropathic pain. Curr. Biol. 16, 1591–1605. 10.1016/j.cub.2006.07.061 16920620

[B49] Regueiro M. Greer J. B. Szigethy E. (2017). Etiology and treatment of pain and psychosocial issues in patients with inflammatory bowel diseases. Gastroenterology 152, 430–439. 10.1053/j.gastro.2016.10.036 27816599

[B50] Ringel-Kulka T. Goldsmith J. R. Carroll I. M. Barros S. P. Palsson O. Jobin C. (2014). Lactobacillus acidophilus NCFM affects colonic mucosal opioid receptor expression in patients with functional abdominal pain - a randomised clinical study. Aliment. Pharmacol. Ther. 40, 200–207. 10.1111/apt.12800 24853043 PMC4613798

[B51] Rizopoulos T. Papadaki-Petrou H. Assimakopoulou M. (2018). Expression profiling of the transient receptor potential vanilloid (TRPV) channels 1, 2, 3 and 4 in mucosal epithelium of human ulcerative colitis. Cells 7, 61. 10.3390/cells7060061 29914124 PMC6025154

[B52] Rogler G. Singh A. Kavanaugh A. Rubin D. T. (2021). Extraintestinal manifestations of inflammatory bowel disease: current concepts, treatment, and implications for disease management. Gastroenterology 161, 1118–1132. 10.1053/j.gastro.2021.07.042 34358489 PMC8564770

[B53] Rousseaux C. Thuru X. Gelot A. Barnich N. Neut C. Dubuquoy L. (2007). Lactobacillus acidophilus modulates intestinal pain and induces opioid and cannabinoid receptors. Nat. Med. 13, 35–37. 10.1038/nm1521 17159985

[B54] Sandborn W. J. Lewis J. D. Panes J. Loftus E. V. D'Haens G. Yu Z. (2022). Association between proposed definitions of clinical remission/response and well-being in patients with Crohn's disease. J. Crohns Colitis 16, 444–451. 10.1093/ecco-jcc/jjab161 34546360 PMC8919823

[B55] Saulnier D. M. Riehle K. Mistretta T. A. Diaz M. A. Mandal D. Raza S. (2011). Gastrointestinal microbiome signatures of pediatric patients with irritable bowel syndrome. Gastroenterology 141, 1782–1791. 10.1053/j.gastro.2011.06.072 21741921 PMC3417828

[B56] Sweeney L. Moss-Morris R. Czuber-Dochan W. Murrells T. Norton C. (2020). Developing a better biopsychosocial understanding of pain in inflammatory bowel disease: a cross-sectional study. Eur. J. Gastroenterol. Hepatol. 32, 335–344. 10.1097/MEG.0000000000001615 31851083

[B57] Tache Y. Larauche M. Yuan P. Q. Million M. (2018). Brain and gut CRF signaling: biological actions and role in the gastrointestinal tract. Curr. Mol. Pharmacol. 11, 51–71. 10.2174/1874467210666170224095741 28240194 PMC8091865

[B58] Takahashi K. Khwaja I. G. Schreyer J. R. Bulmer D. Peiris M. Terai S. (2021). Post-inflammatory abdominal pain in patients with inflammatory bowel disease during remission: A comprehensive review. Crohns Colitis 360, otab073. 10.1093/crocol/otab073 PMC9802269 36777266

[B59] van Hoboken E. A. Thijssen A. Y. Verhaaren R. van der Veek P. P. Prins F. A. Verspaget H. W. (2011). Symptoms in patients with ulcerative colitis in remission are associated with visceral hypersensitivity and mast cell activity. Scand. J. Gastroenterol. 46, 981–987. 10.3109/00365521.2011.579156 21623672

[B60] van Thiel I. Botschuijver S. de Jonge W. J. Seppen J. (2020). Painful interactions: Microbial compounds and visceral pain. Biochim. Biophys. Acta Mol. Basis Dis. 1866, 165534. 10.1016/j.bbadis.2019.165534 31634534

[B61] Vermeulen W. De Man J. G. Pelckmans P. A. De Winter B. Y. (2014). Neuroanatomy of lower gastrointestinal pain disorders. World J. Gastroenterol. 20, 1005–1020. 10.3748/wjg.v20.i4.1005 24574773 PMC3921524

[B62] Wallon C. Yang P. C. Keita A. V. Ericson A. C. McKay D. M. Sherman P. M. (2008). Corticotropin-releasing hormone (CRH) regulates macromolecular permeability via mast cells in normal human colonic biopsies *in vitro* . Gut 57, 50–58. 10.1136/gut.2006.117549 17525093

[B63] Wan Y. Shen J. Ouyang J. Dong P. Hong Y. Liang L. (2022). Bibliometric and visual analysis of neutrophil extracellular traps from 2004 to 2022. Front. Immunol. 13, 1025861. 10.3389/fimmu.2022.1025861 36341351 PMC9634160

[B64] Wang H. Shi J. Shi S. Bo R. Zhang X. Hu Y. (2022). Bibliometric analysis on the progress of chronic heart failure. Curr. Probl. Cardiol. 47, 101213. 10.1016/j.cpcardiol.2022.101213 35525461

[B65] Wils P. Caron B. D'Amico F. Danese S. Peyrin-Biroulet L. (2022). Abdominal pain in inflammatory bowel diseases: A clinical challenge. J. Clin. Med. 11, 4269. 10.3390/jcm11154269 35893357 PMC9331632

[B66] Wu S. V. Yuan P. Q. Lai J. Wong K. Chen M. C. Ohning G. V. (2011). Activation of type 1 CRH receptor isoforms induces serotonin release from human carcinoid BON-1N cells: an enterochromaffin cell model. Endocrinology 152, 126–137. 10.1210/en.2010-0997 21123435 PMC3219048

[B67] Zhou Q. Nicholas V. G. (2008). NMDA receptors and colitis: Basic science and clinical implications. Rev. Analg. 10, 33–43. 10.3727/154296108783994013 20574552 PMC2889709

[B68] Zorina-Lichtenwalter K. Meloto C. B. Khoury S. Diatchenko L. (2016). Genetic predictors of human chronic pain conditions. Neuroscience 338, 36–62. 10.1016/j.neuroscience.2016.04.041 27143481

