# Supplementary figures and images for: Research hotspots and trend analysis of abdominal pain in inflammatory bowel disease: a bibliometric and visualized analysis
Source: Front Pharmacol. 2023 Sep 21;14:1220418. doi: 10.3389/fphar.2023.1220418 (PMC10552780; doi:10.3389/fphar.2023.1220418)

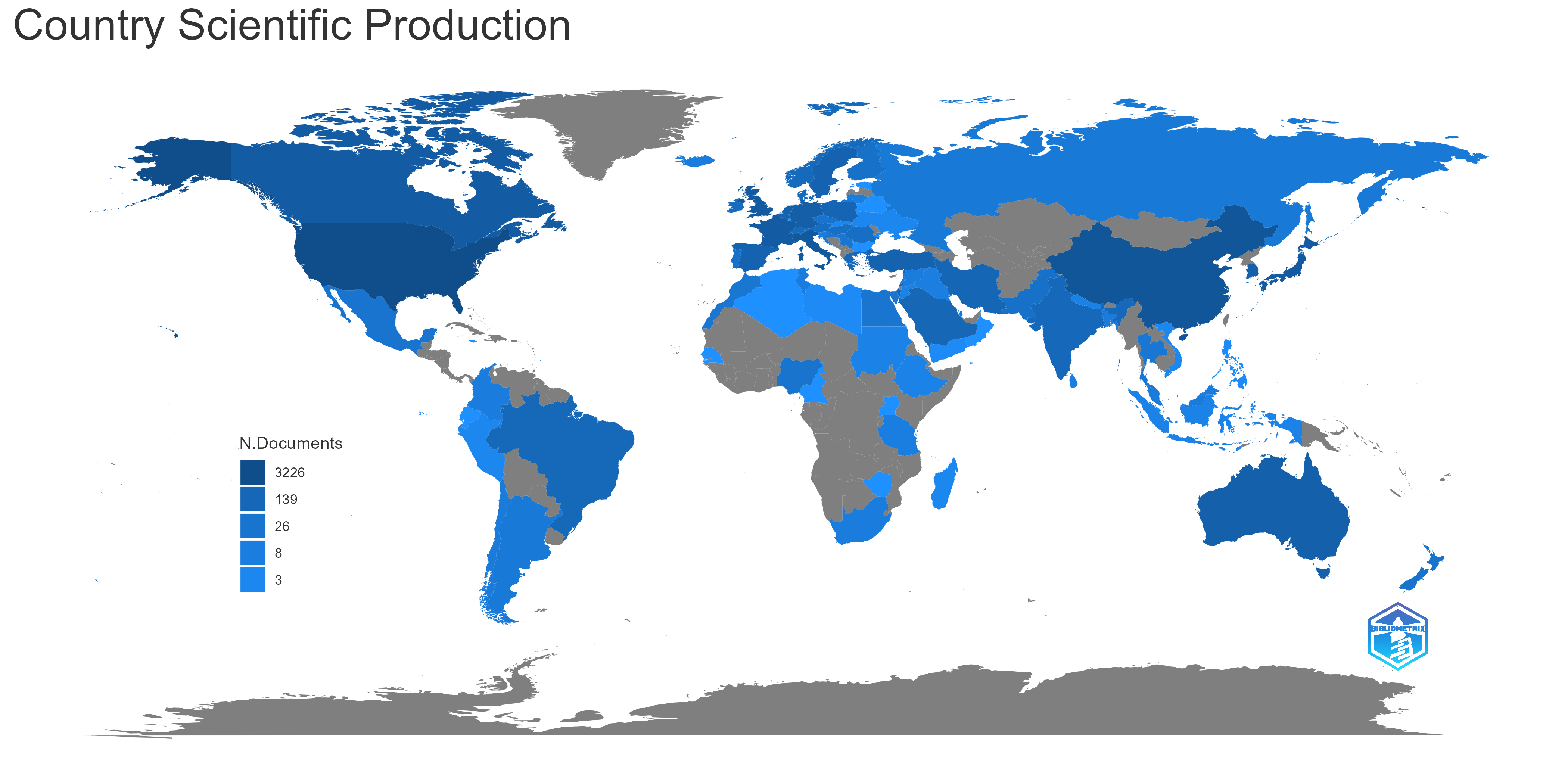

Supplement: Supplementary file 1 [file DataSheet1.zip › Supplementary Material/S1-CountryScientificProduction-2023-04-22.png]

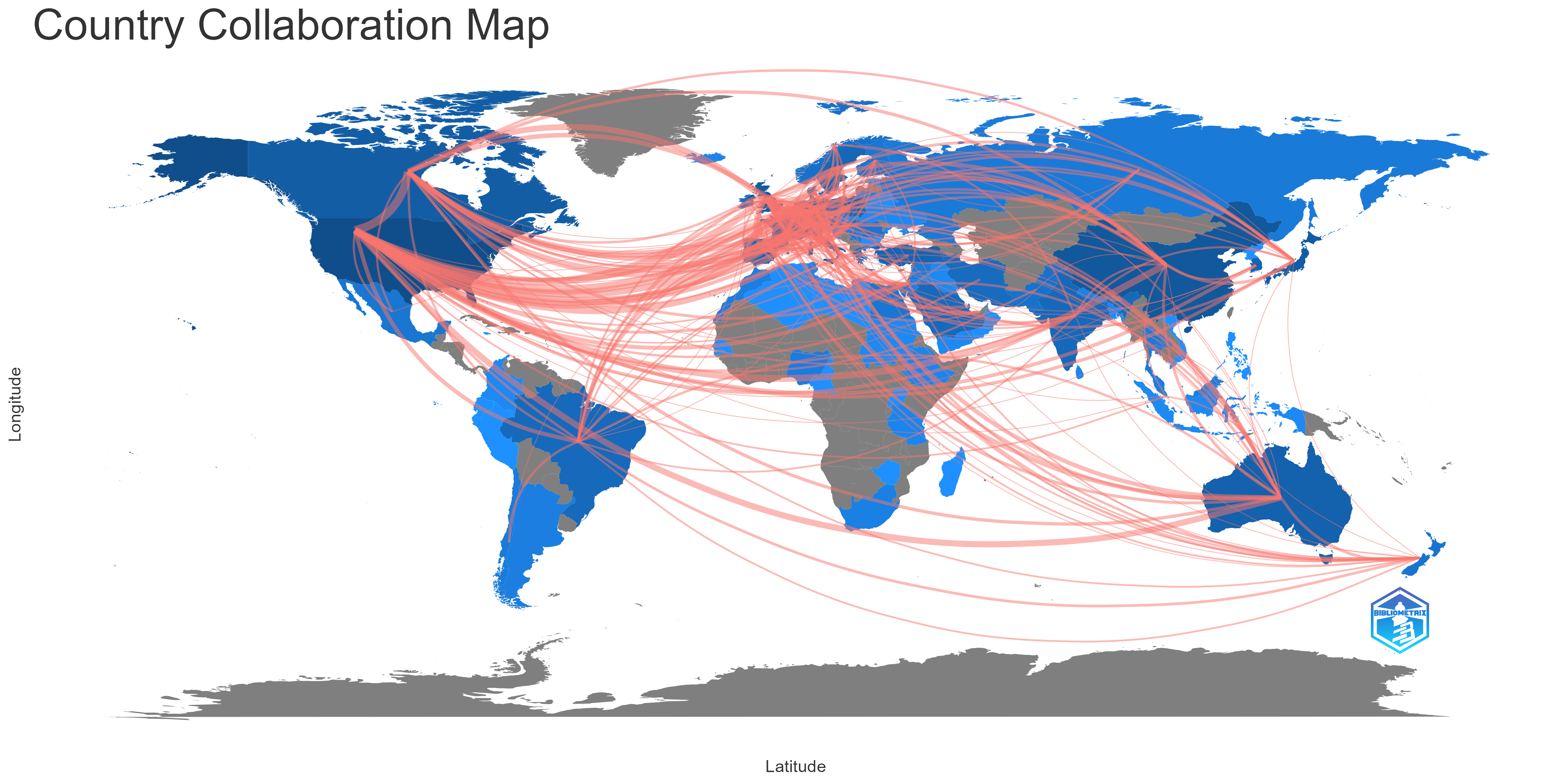

Supplement: Supplementary file 1 [file DataSheet1.zip › Supplementary Material/S2-CountryCollaborationMap-2023-04-22.png]

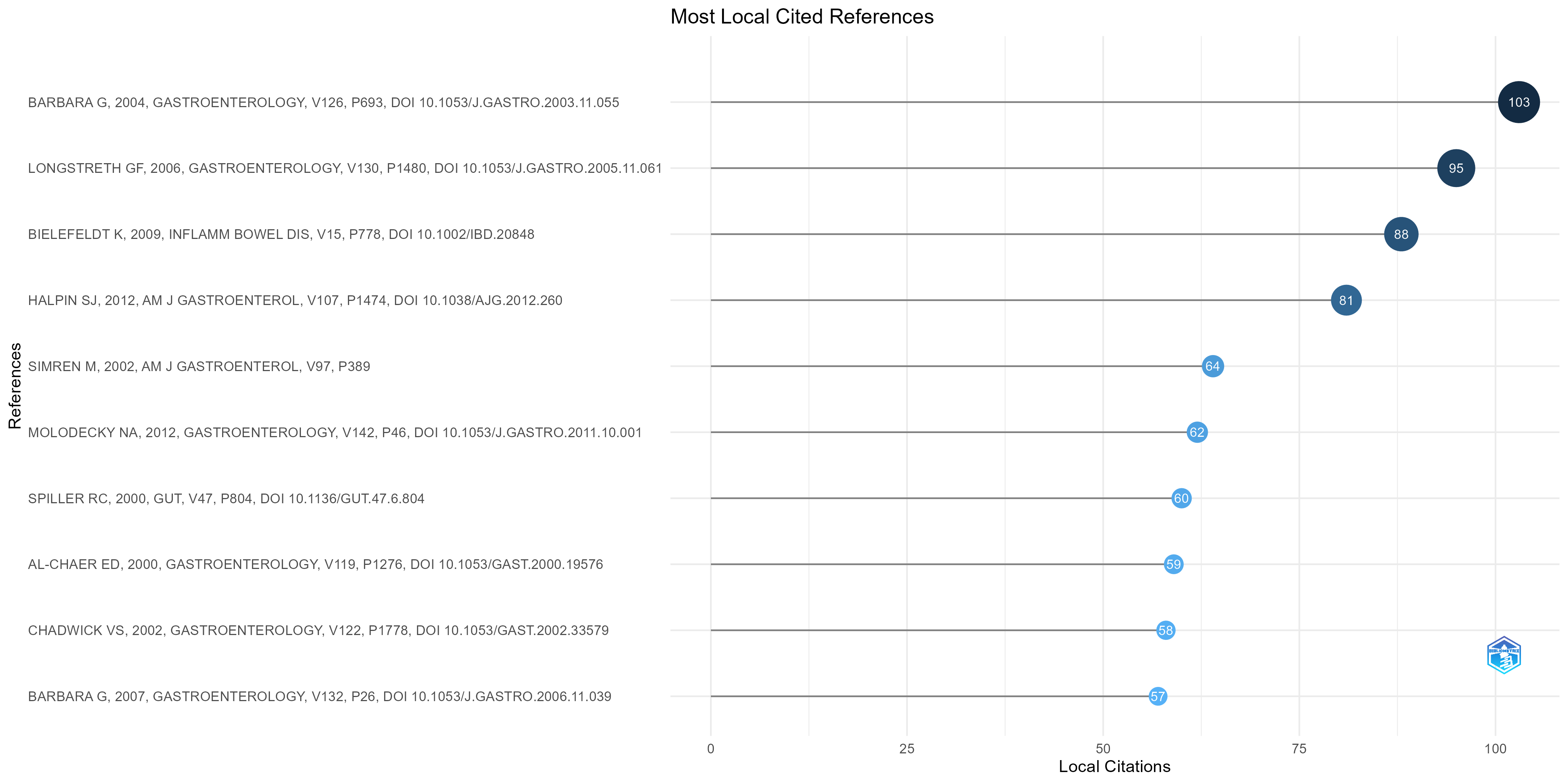

Supplement: Supplementary file 1 [file DataSheet1.zip › Supplementary Material/S3-MostLocalCitedReferences-2023-04-22.png]

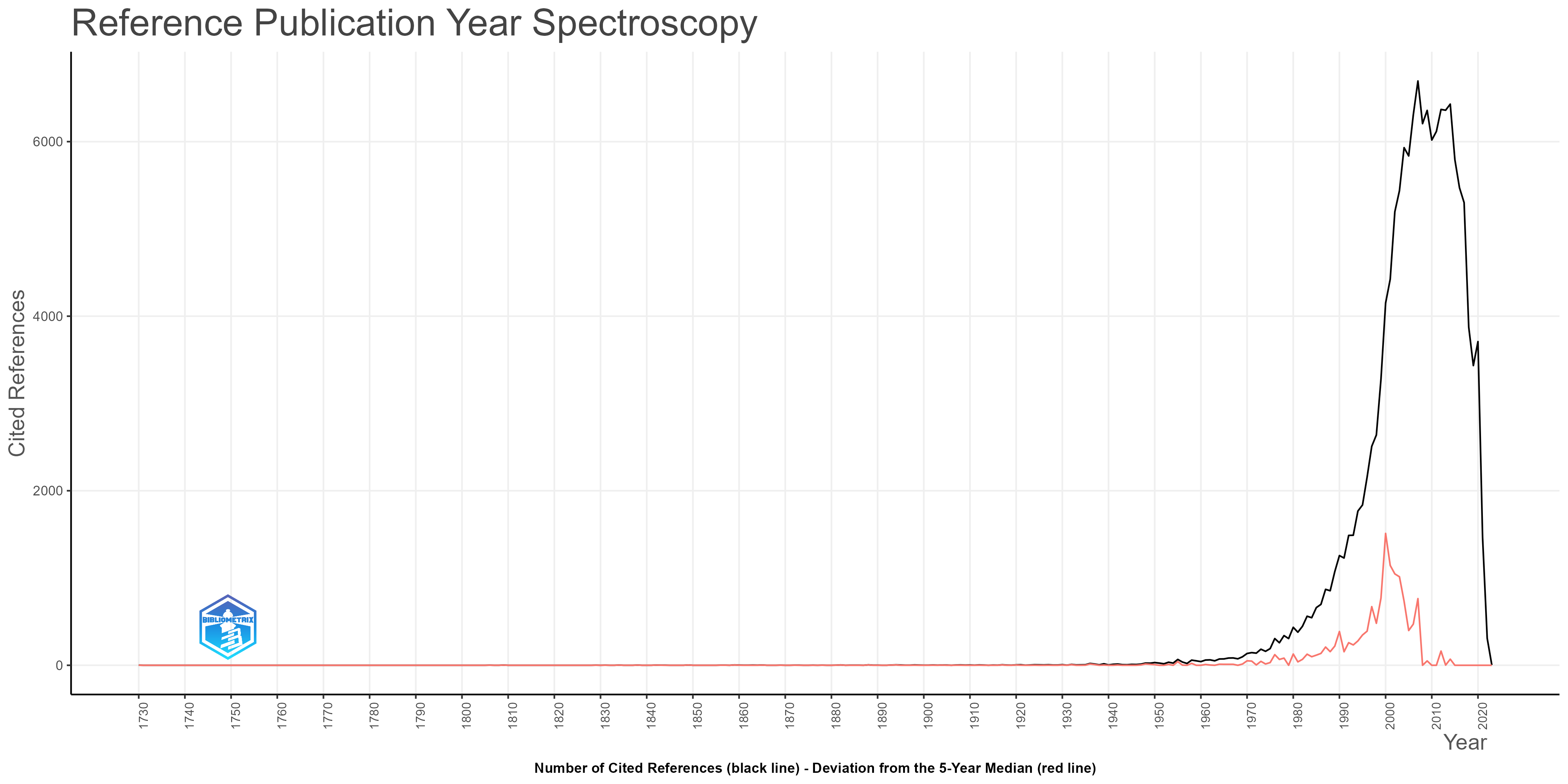

Supplement: Supplementary file 1 [file DataSheet1.zip › Supplementary Material/S4-ReferenceSpectroscopy-2023-04-22.png]

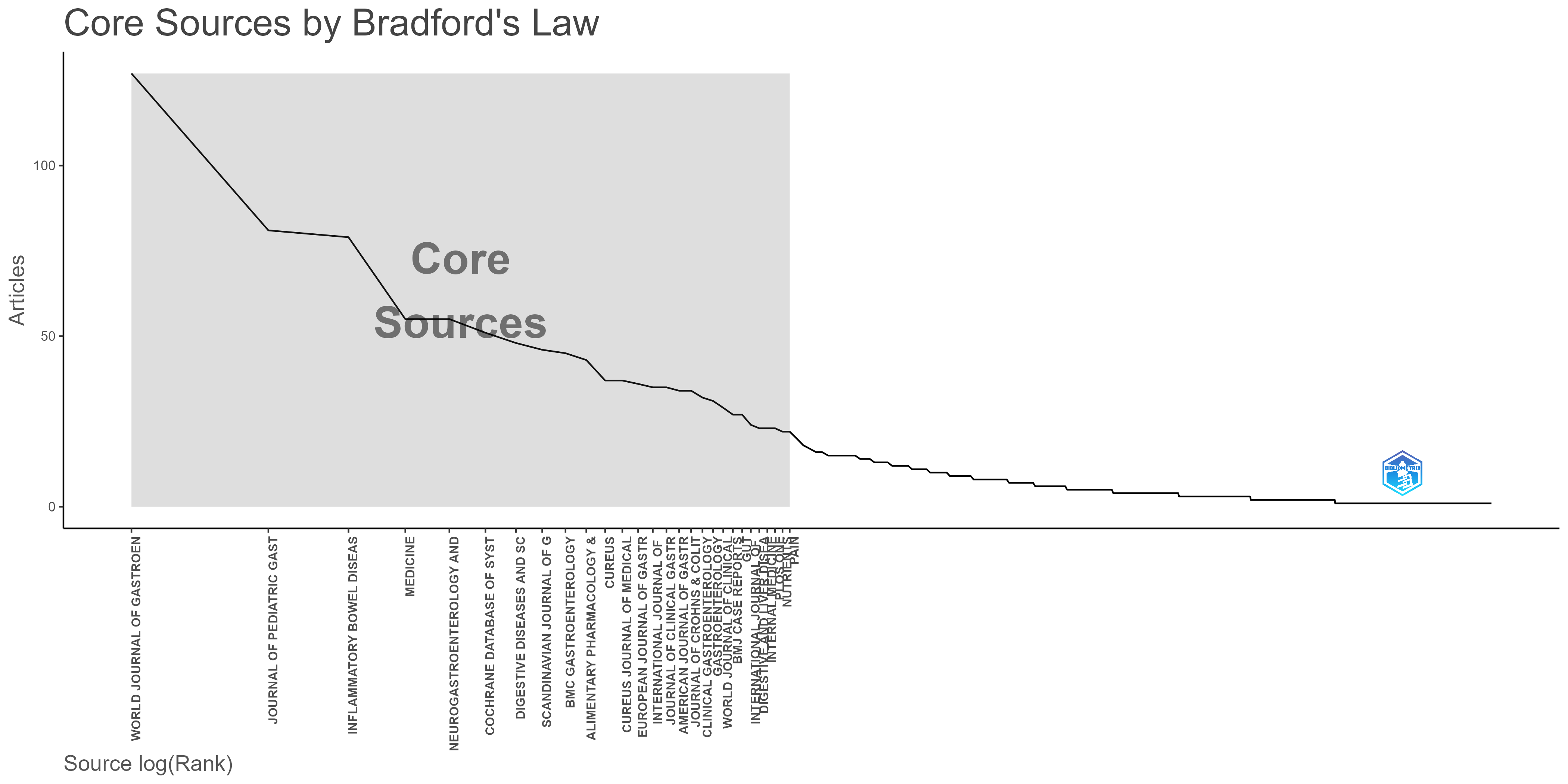

Supplement: Supplementary file 1 [file DataSheet1.zip › Supplementary Material/S5-BradfordLaws-2023-04-22.png]
